# Modulation of Notch Signaling at Early Stages of Differentiation of Human Induced Pluripotent Stem Cells to Dopaminergic Neurons

**DOI:** 10.3390/ijms24021429

**Published:** 2023-01-11

**Authors:** Nataliia V. Katolikova, Aleksandr A. Khudiakov, Daria D. Shafranskaya, Andrey D. Prjibelski, Alexey E. Masharskiy, Mikael S. Mor, Alexey S. Golovkin, Anastasia K. Zaytseva, Irina E. Neganova, Evgeniya V. Efimova, Raul R. Gainetdinov, Anna B. Malashicheva

**Affiliations:** 1 Institute of Translational Biomedicine, Saint-Petersburg State University, 199034 Saint-Petersburg, Russia; 2 Almazov National Medical Research Centre, 197341 Saint-Petersburg, Russia; 3 Center for Algorithmic Biotechnology, Institute of Translational Biomedicine, Saint-Petersburg State University, 199034 Saint-Petersburg, Russia; 4 Resource Center “Bio-Bank Center”, Research Park of Saint-Petersburg State University, 198504 Saint-Petersburg, Russia; 5 Institute of Cytology, Russian Academy of Sciences, 194064 Saint-Petersburg, Russia; 6 Saint-Petersburg University Hospital, 199034 Saint-Petersburg, Russia

**Keywords:** induced pluripotent stem cells (IPSC), dopamine neurons, differentiation, *RBPJ*, DAPT, notch intracellular domain (NICD), Parkinson’s disease

## Abstract

Elaboration of protocols for differentiation of human pluripotent stem cells to dopamine neurons is an important issue for development of cell replacement therapy for Parkinson’s disease. A number of protocols have been already developed; however, their efficiency and specificity still can be improved. Investigating the role of signaling cascades, important for neurogenesis, can help to solve this problem and to provide a deeper understanding of their role in neuronal development. Notch signaling plays an essential role in development and maintenance of the central nervous system after birth. In our study, we analyzed the effect of Notch activation and inhibition at the early stages of differentiation of human induced pluripotent stem cells to dopaminergic neurons. We found that, during the first seven days of differentiation, the cells were not sensitive to the Notch inhibition. On the contrary, activation of Notch signaling during the same time period led to significant changes and was associated with an increase in expression of genes, specific for caudal parts of the brain, a decrease of expression of genes, specific for forebrain, as well as a decrease of expression of genes, important for the formation of axons and dendrites and microtubule stabilizing proteins.

## 1. Introduction

Effective and specific protocols for differentiation of human pluripotent stem cells, either human embryonic stem (hES) cells or human induced pluripotent stem (hIPS) cells, to dopaminergic (DA) neurons and their precursors are very important for development of cell replacement therapy of Parkinson’s disease, and, to date, such protocols have already been developed by a number of scientific groups [1,2,3]. Most of them start with neuronal induction by the double SMAD inhibition [4], continue with further regionalization of neuronal differentiation towards DA neurons through activation of SHH, WNT and FGF8 signaling pathways and terminate through maturation of obtained neuronal precursors in the presence of neuronal growth factors. However, a number of other signaling pathways, important for neurogenesis, may also contribute to the specificity and efficiency of the protocols and studying of their modulation can give deeper understanding of their role in neuronal development.

Notch signaling plays an extremely essential role both during embryonic development of the central nervous system and postnatally, taking part in maintaining the pool of stem cells, regulation of proliferation, differentiation, migration and apoptosis [5,6,7].

The Notch pathway consists of several ligands (Delta-like1, Delta-like3, Delta-like4 and Jagged 1, Jagged 2) and receptors (Notch1, Notch2, Notch3, Notch4). Interaction between a ligand and a receptor activates Notch intracellular domain (NICD) cleavage by gamma-secretase and follows by NICD binding to the transcriptional factor CSL encoded by an *RBPJ* gene to regulate downstream Notch-dependent genes [8].

Non-lethal mutations in genes encoding for Notch receptors and ligands lead in particular to a disruption of cell migration during embryonic development, for instance, migration of neuronal progenitor cell into the cortex [9].

Activation of Notch signaling cascade has been described in regions of the adult brain, which are important for adult neurogenesis: subventricular and subgranular zones [6]. Non-lethal mutations of Notch receptors or mutations of *RBPJ*, which provides signaling from all four Notch receptors, lead to impaired survival of neuronal progenitors which in turn results in a sharp decrease of their amount. In an adult organism, it leads to the loss of neuronal stem cells, premature differentiation and, thus, the depletion of the neuronal stem cell pool [7]. RBPJ is known to control neurogenesis by maintaining neural progenitor cells and inhibiting their premature differentiation into neurons [5]. On the other hand, RBPJ plays an important role in the formation of the cortex being required for correct positioning of cortical neurons through the regulation of neuronal migration and safekeeping of radial glial scaffolds [5]

In vitro studies demonstrated that neuronal progenitors derived from ES cells express all components of the Notch pathway: ligands, receptors, and target genes. Activated Notch signaling is required to maintain neuronal progenitors in an undifferentiated/self-renewing state [10]. Inhibition of Notch signaling plays a role in the differentiation of neurospheres into DA neurons. Tieng at al. showed that inhibition of gamma secretase, which causes downregulation of Notch signaling, during differentiation increases the number of tyrosine hydroxylase (TH) positives, markers of DA neurons, and neurons in obtained cell cultures [11].

However, the effects of the Notch signaling modulation are not always univocal. The consequence of activating or inhibiting of Notch signaling is extremely dependent on environment. Notch signaling activity is closely related to the state of the Wnt signaling cascade. Inhibition of Notch signaling by N-[N-(3,5-difluorophenacetyl)-l-alanyl]-s-phenylglycinet-butyl ester (DAPT), gamma-secretase inhibitor, accompanied with inhibition of Wnt signaling promotes an increase in the number of TUJ (neuron-specific class III beta-tubulin), neuronal marker, and positive cells during differentiation of pluripotent stem cells into cortical neurons. Under the same conditions, but with activation of Wnt signaling, the number of TUJ-positive neurons becomes significantly lower [12]. Small molecule activator of Wnt signaling CHIR99021 increased the expression of Notch downstream target HES1 irrespectively of the DAPT treatment, suggesting that WNT signaling could dominate over the Notch [13].

Taking into account the meaning role, which Notch signaling plays in the nervous system development, it is important to understand what the consequences of Notch modulation are during the differentiation of pluripotent stem cells towards various directions. In our study, we have investigated the role of inhibition of Notch signaling during the first 7 days of differentiation of hIPS cells to DA neurons—wherein we compared two variants of inhibition of Notch signaling: by gamma-secretase inhibitor DAPT, which is the most common agent for Notch signaling inhibition, and by gene-specific short hairpin RNA (shRNA) to *RBPJ*, the downstream target of Notch signaling. Gamma-secretase has other targets besides the Notch receptor such as E-cadherin, N-cadherin, ephrin-B2, CD44 and amyloid beta precursor protein, so this method does not represent a highly selective inhibition of the Notch signaling pathway. On the other hand, we investigated the role of activation of Notch signaling by overexpression of *NICD* during the first 7 days of differentiation of hIPS cells to DA neurons.

## 2. Results

### 2.1. Dopaminergic Neurons Were Efficiently Generated from hIPS Cell Lines

For generation of midbrain DA neurons from three lines of hIPS cells (AD3, WTSIi004-A, WTSIi046-A), we used protocol, published in Doi et al. [1] with modification (Figure S1A).

Using immunofluorescent staining, we confirmed that undifferentiated hIPS cells expressed two pluripotency markers OCT4 and NANOG (Figure S1B).

After 53 days of differentiation, obtained cells acquired morphology typical for neurons. Immunofluorescent analysis showed that, at this stage, the overwhelming majority of the cells expressed markers of mature dopaminergic neurons: TH, MAP2 (Figure S1C). Flow cytometry analysis demonstrated that most of the cells (approximately 90%) were positive for TH on day 53 of differentiation (Figure S1D). High performance liquid chromatography showed that cells contained dopamine and its metabolites DOPAC and homovanillic acid (HVA) (Figure S1F). However, it should be noted that we detected a small amount of serotonin and its metabolite 5-hydroxyindoleacetic acid (5-HIAA), indicating that the resulting cell culture consisted not only of dopaminergic neurons, but, probably, contained a small number of serotonergic neurons. The electrophysiological parameters of the resulting cell culture were evaluated using the patch clamp method. Obtained neurons were able to spontaneously generate action potentials (AP) with a typical shape for neuronal cells with pronounced phases of depolarization and repolarization (Figure S1E).

Thus, we confirmed that the protocol we used is effective for obtaining mature dopaminergic neurons culture in vitro.

### 2.2. RBPJ Knockdown and NICD Overexpression Are Effective Approaches for Notch Signaling Inhibition/Activation Respectively during hIPS Cells Differentiation to DA Neurons

**Figure 1:**
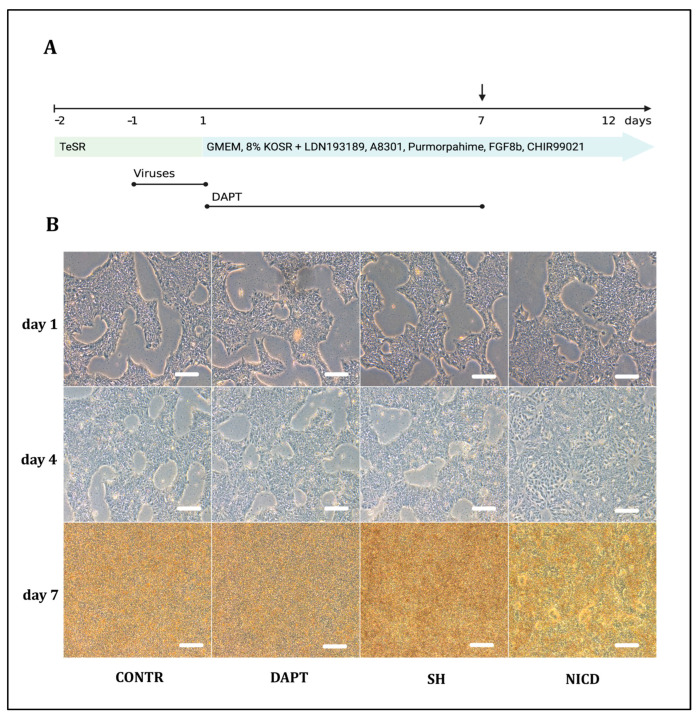
Modulation of Notch signaling during early stages of differentiation of human induced pluripotent stem (hIPS) cells to dopaminergic (DA) neurons. (**A**) the experiment scheme; viral particles were added on the next day after cell seeding and incubated for 24 h. DAPT was added from days 1 to 7 of differentiation. Cells were taken for RNA isolation on day 7 of differentiation. Created in BioRender.com; (**B**) transmitted light, 4 conditions: CONTR, non-treated cells; DAPT, cells treated with DAPT; SH, cells with *RBPJ* knockdown; NICD, cells with *NICD* overexpression. Morphological changes in cell culture on days 1, 4, 7 of differentiation of hIPS cells to DA neuron with modulation of Notch signaling showed similarity in CONTR, SH and DAPT conditions and differences in NICD condition, which manifested with the formation of rosette like structures starting from day 4. Scale bars 200 μm.

To inhibit Notch signaling, we performed *RBPJ* knockdown by lentiviral transduction of shRNA or used gamma-secretase inhibitor DAPT treatment from days 1 to 7 (Figure 1A). To activate Notch signaling, we performed lentiviral transduction of hIPS cells with lentiviral particles carrying *NICD* gene coding for Notch intracellular domain. Taken together, we used four different conditions in our experiment: CONTR, non-treated cells; SH, cells with *RBPJ* knockdown; NICD, cells with *NICD* overexpression; DAPT, cells treated with DAPT (Table 1).

We analyzed the efficiency of inhibition and activation of Notch signaling by *RBPJ* knockdown and overexpression of *NICD*, respectively, after 96 h of transduction and on day 7 of differentiation and showed that *RBPJ* knockdown reduced the expression of *RBPJ*, the downstream target of the Notch signaling, to 30% of control levels (Figure S2). Overexpression of *NICD* increased *HEY1* expression, the downstream target of the Notch signaling, by 36 times compared to the control condition. This effect was observed at 96 h after transduction and persisted throughout the entire time until the end of the experiment (7 days of differentiation).

Changes in cell morphology during 7 days of differentiation were similar in the CONTR, SH, DAPT groups (Figure 1B). Cells overexpressing *NICD* were noted to acidify the medium much faster in comparison with other conditions and to form rosette-like structures starting from day 4.

### 2.3. Analysis of RNA Sequencing Data by Principal Component Analysis (PCA) Revealed the Similarities and Differences between the Groups

**Figure 2:**
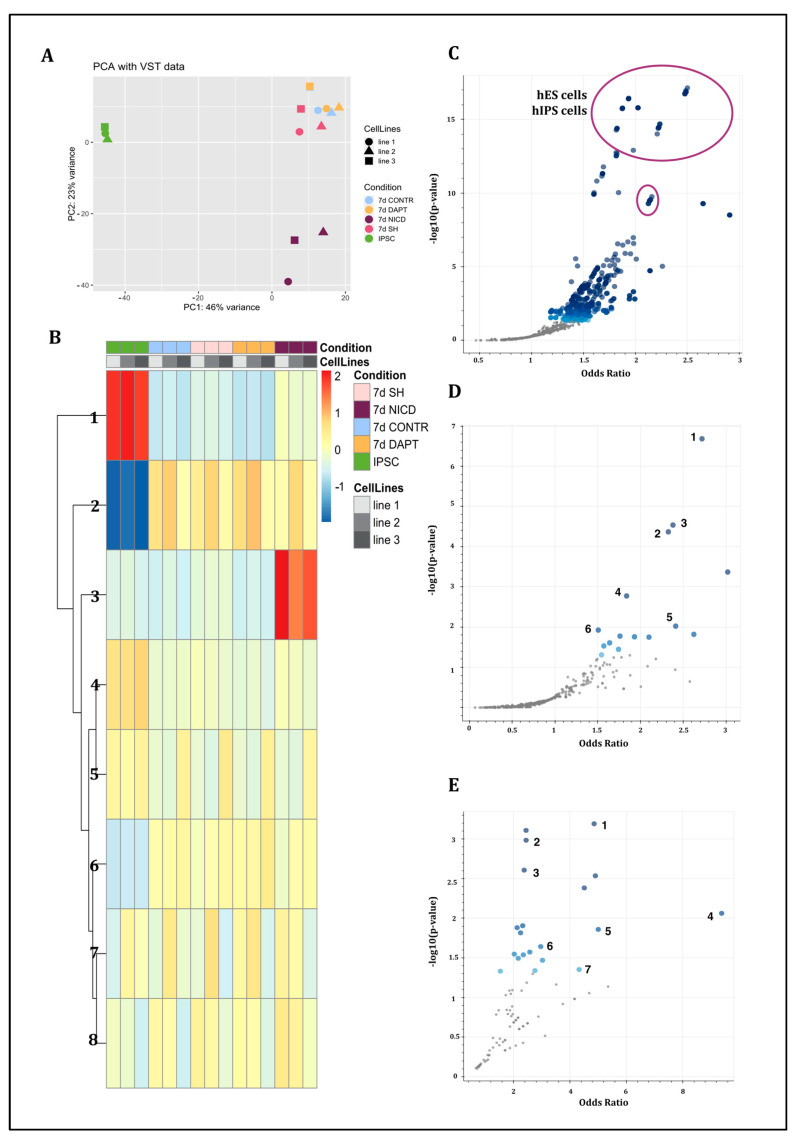
Analysis of RNA sequencing data. (**A**) principal component analysis of non-treated cells (CONTR), cells with *RBPJ* knockdown (SH), cells with *NICD* overexpression (NICD), cells treated with DAPT (DAPT) showed similarities of CONTR, SH and DAPT condition, and significant differences in NICD condition. (**B**) Cluster analysis of CONTR, SH, NICD, DAPT conditions showed division into seven clusters. (**C**–**E**) volcano plots depicting odds ratios and *p*-values of gene expression levels within selected clusters; (**C**) group of genes from cluster 1 of cluster analysis showed the presence of genes, characteristic for different lines of hIPS cells and hES cells (in circles); (**D**) group of genes from cluster 2 of cluster analysis showed the presence of genes, characteristic for (1) axon guidance (2) Wnt signaling pathway (3) Hippo signaling pathway (4) focal adhesion (5) Hedgehog signaling pathway (6) MAPK signaling pathway; (**E**) group of genes from cluster 3 of cluster analysis showed the presence of genes, characteristic for (1) Notch signaling pathway (2) Integrin signaling pathway (3) angiogenesis (4) pyruvate metabolism (5) opioid proopiomelanocortin pathway (6) ubiqitine proteasome pathway (7) GABA-B receptor II signaling.

RNA sequencing was performed on day 7 of differentiation. Principal component analysis (Figure 2A) revealed significant differences between NICD and CONTR, and NICD and SH groups. No significant differences were observed between CONTR and SH as well as between CONTR and DAPT groups. Different cell lines within the same experimental condition clustered together.

### 2.4. Cluster Analysis Identified Groups of Genes Characteristic for hIPS Cells, for Differentiated Conditions and in Addition for NICD Condition

For cluster analysis, we used the transcriptomic data obtained on the 7th day of differentiation in all experimental groups, as well as the data of RNA sequencing of the non-differentiated hIPS cells on day 0 (Figure 2B).

It was found that the expression of pluripotent stem cell-specific genes decreased by day 7 of differentiation, regardless of the presence or absence of Notch signaling cascade modulation (Cluster 1 Figure 2B; Figure 2C; Table S1).

During differentiation expression of genes related to SHH, Wnt and MAPK signaling cascades, as well as genes regulating nerve cell outgrowths, in particular axon formation, was increased (Cluster 2 Figure 2B; Figure 2D; Table S1).

Using cluster analysis, we identified a separate group of genes that distinguished the NICD group from all the other conditions. This group included genes associated with the receptor–ligand interaction; activity of kinases or protein binding to kinases and including metabolism-related genes (Cluster 3 Figure 2B; Figure 2E; Table S1).

### 2.5. Differential Expression Analyses Showed Predominant Similarities of Control Differentiation (CONTR Group) and Differentiation with Inhibition of Notch Signaling (SH and DAPT Groups) and Significant Changes Compared with Differentiation under Activation of Notch Signaling (NICD Group)

#### 2.5.1. Comparison of CONTR and NICD Groups

**Figure 3:**
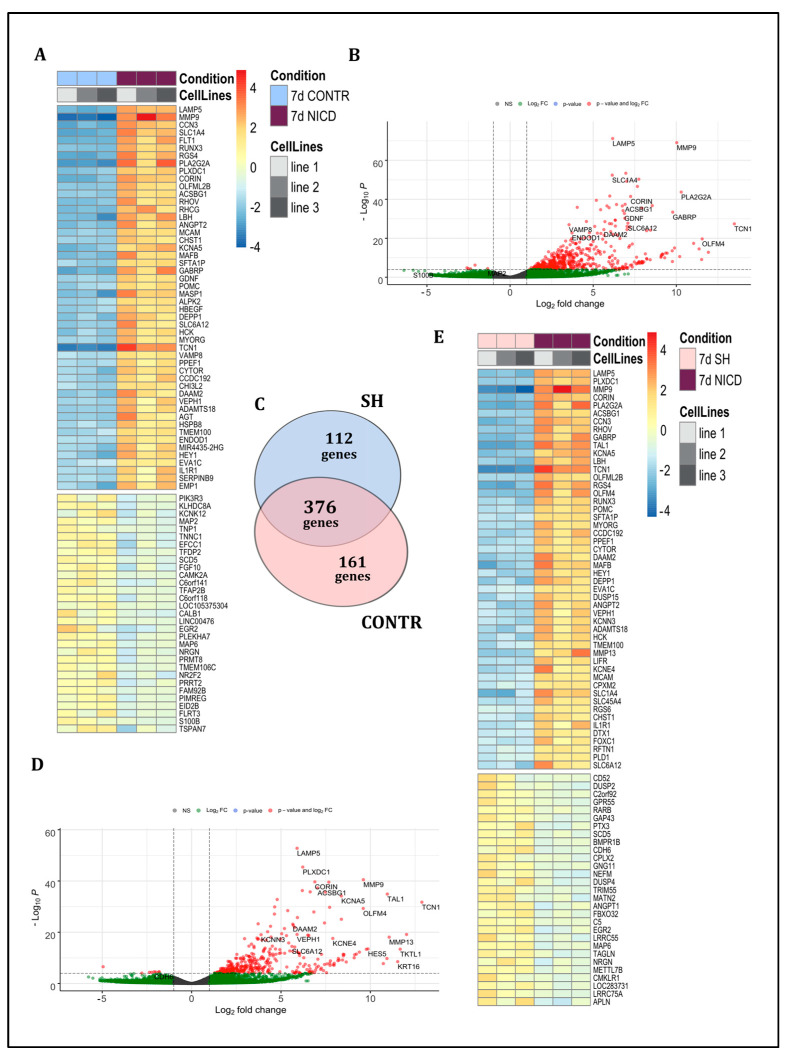
Comparison of transcriptomic profiles of non-treated cells (CONTR) with cells with *NICD* overexpression (NICD), and cells with *RBPJ* knockdown (SH) with NICD. (**A**) The heat map of differentially expressed genes between CONTR and NICD conditions showed an increased expression of *DAAM2, ACSBG1, CORIN, GDNF, SLC6A12* genes, and a decrease of *CALB1, CAMK2A, NRGN, PIK3R3, MAP2, MAP6, S100B, TSPAN7* genes in NICD conditions, top 50 genes. (**B**) volcano plot of differentially expressed genes between groups CONTR and NICD conditions; (**C**) Venn diagram of overexpressed genes in the NICD group relative to SH and CONTR groups showed the dominant overlap between these two groups; (**D**) volcano plot of differentially expressed genes between SH and NICD conditions showed an increase of expression of *DAAM2, ACSBG1, VEPH1, CORIN* in NICD conditions; (**E**) heat map of differentially expressed genes between SH and NICD conditions, top 50 genes.

By comparing the two sets of data, we showed that overexpression of NICD during the first 7 days of differentiation to DA neurons resulted in upregulation of a number of genes (Figure 3A,B; Table S2). Among these genes, the prevailing majority was directly related to the regulation of the nervous system development and the neuronal specification. In particular, in the NICD group, the expression of genes characteristic for the development of the thalamus (*ENDOD1, SLC1A4*) and spinal cord (*DAAM2, ACSBG1*) was increased, whereas expression of markers characteristic for pyramidal neurons (*CALB1, MAP2, CAMK2A, NRGN*), GABAergic neurons (*MAP2, CAMK2A, MAP6*), interneurons (*CALB1, MAP2, CAMK2A, NRGN*), as well as cholinergic neurons (*CAMK2A, PIK3R3*), was reduced.

In the NICD group, we have found the decrease of expression of *S100B* and *TSPAN7*, genes important for axon growth, and the *TSPAN7* gene known to be critical for cerebellar granule cell axonal branching [14]. In the NICD group, we observed the decrease of *MAP6* and *MAP2* involved in microtubule stabilization.

We have shown an increase of expression of *CORIN* in the NICD group. CORIN is used as a marker for DA neuron precursors [1]. In addition, we found the increased expression of the glial neurotrophic factor (GDNF).

We described an increased expression of several types of ion channels in the NICD group: *KCNA5*, a voltage-gated potassium ion channel playing a role in neuronal excitability, release of neurotransmitters and involved in regulation of potassium current in human atrial; and *GABRP*, the gamma-aminobutyric acid receptor (GAMK) type A, which is an ion channel for chloride and plays an important role in inhibitory synaptic transmission.

In this condition, we found an increase of the expression of *DAAM2*, an activator of Wnt signaling, as well as *VEPH1*, which inhibits SMAD proteins, through interaction with TGF-beta receptor type-1 (TGFBR1) receptors.

NICD group was also found to have an increased expression of *SLC6A12*, a betaine-GABA transporter that works in presynaptic terminals, and *VAMP8*, which is actively involved in the fusion of synaptic vesicles with the presynaptic membrane.

#### 2.5.2. Comparison of SH and NICD Groups

PCA analysis of transcriptomic data showed the overlap between SH and CONTR groups (Figure 2A). Taking this into account, we expected that the results of SH and NICD comparison would be similar to these of the CONTR and NICD groups. Indeed, among the overexpressed genes in the NICD group, evaluated in relation to the CONTR or to the SH, 376 genes were common to both comparisons (Figure 3C). Significant similarity was also preserved between the top 50 overexpressed genes: 36 genes out of top 50 were common to both comparisons (Figure S3).

Activation of the Notch signaling pathway was associated with upregulation of the spinal cord marker, such as *DAAM2*, which can bind to actin and small GTPases and affects Wnt signaling, and *ACSBG1*, which plays an important role in the formation of the myelin sheath (Figure 3D,E; Table S2).

We found upregulation of *VEPH1* and downregulation of *CDH6* in the NICD group, which cause the inhibition of TGF beta and BMP signaling pathways. We also found upregulation of *DAAM2* associated with activation of SHH and Wnt signaling.

In addition, in the NICD group, we detected an upregulation of the *CORIN* gene known as a marker of DA neuron progenitors.

The NICD group was also characterized by affected synaptic plasticity and neuronal precursor activity manifesting through the increasing expression of *KCNA5*, *KCNN3*, and *KCNE4* potassium channels playing a role in the neurotransmitters release and downregulation of *LRRC55*, a protein, which modulates gating properties of calcium-activated and voltage-dependent potassium channels.

The expression of transporters for betaine with GABA—SLC1A4 and SLC6A12 was also increased in the NICD group. However, the expression of *NEFM*, a neurofilament associated protein, and *MAP6*, a microtubule associated protein, was decreased.

#### 2.5.3. Comparison of CONTR and SH Groups

**Figure 4:**
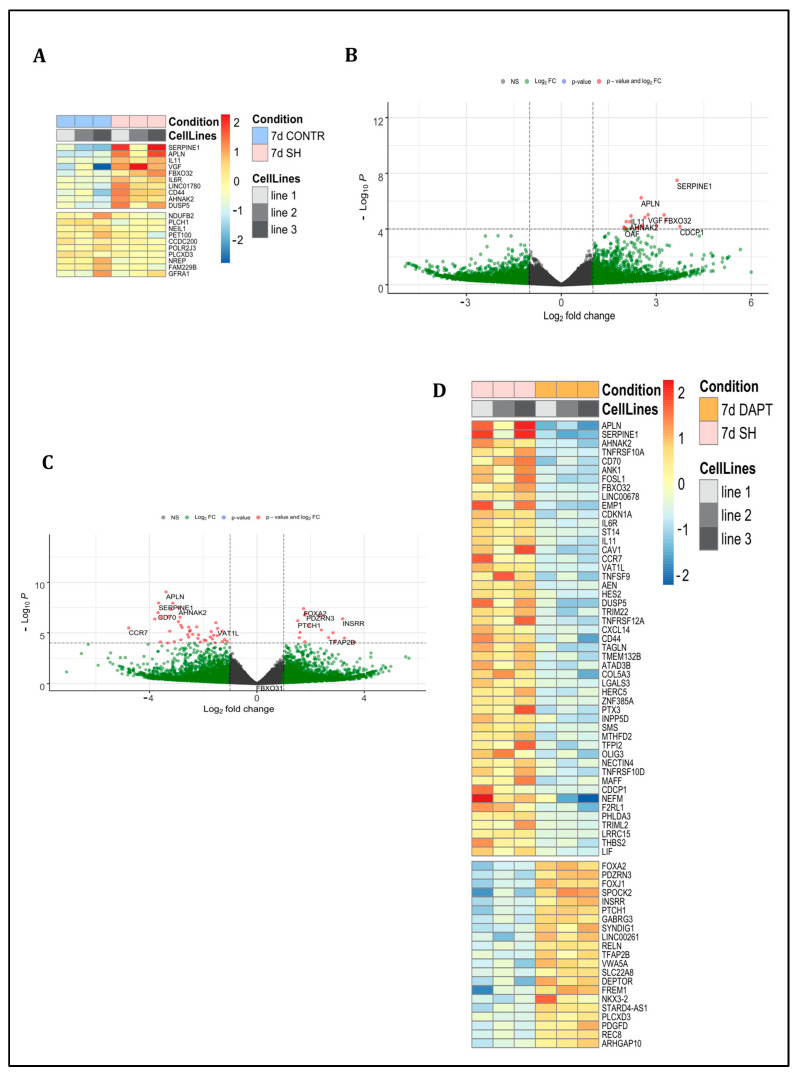
Pairwise comparison of RNA sequencing data of non-treated cells (CONTR) to cells with *RBPJ* knockdown (SH), and SH cells to cells treated with DAPT (DAPT). (**A**) A heat map of differentially expressed genes between CONTR and SH conditions showed a low number of genes with different expression, top 20 genes. (**B**) Volcano plot of differentially expressed genes between CONTR and SH conditions showed only a few genes with increased expression in the SH group. (**C**) Volcano plot of differentially expressed genes between SH and DAPT conditions showed increased expression of *PTCH1, FOXA2, DEPTOR* genes in the DAPT condition. (**D**) The heat map of differentially expressed genes between groups SH and DAPT conditions, top 50 genes.

We analyzed the difference between the first 7 days of hIPS cells differentiation into DA neurons under the CONTR condition and under the SH condition (Figure 4A,B; Table S2). According to the RNA sequencing data, we did not find significant differences between these two conditions.

#### 2.5.4. Comparison of SH and DAPT Groups

In our study, we compared the effects of Notch inhibition by the *RBPJ* knockdown with gamma-secretase inhibition by DAPT treatment (Figure 4C,D; Table S2).

We found that Notch inhibition by DAPT led to a more active state of SHH signaling due to higher expression of the *PTCH1*. In the DAPT group, we also found activation of Wnt signaling and a more active state of the KRAS signaling cascade. In addition, the DAPT group was characterized by the higher expression of *DEPTOR*, a negative mTOR signaling regulator, in comparison with the SH group.

## 3. Discussion

Elaboration of effective and specific protocols for differentiation of human pluripotent stem cells to DA neurons or their precursors is an important issue for the development of cell replacement therapy of Parkinson’s disease. Investigation of the role of modulation of certain signaling pathways may contribute to the deeper understanding of their role in neuronal development and to improving specificity and efficiency of differentiation protocols.

Notch signaling plays an essential role in development of the central nervous system and in the functioning of zones of adult neurogenesis after birth [5,6,7].

In our work, we analyzed how activation and inhibition of Notch signaling influence the early stages of differentiation of hIPS cells into DA neurons. We used the protocol, the first part of which is based on double SMAD inhibition with regionalization of neuronal differentiation towards DA neurons through activation of SHH, WNT, and FGF8 signaling pathways [1]. We modulated Notch signaling during the first 7 days of differentiation. We compared two approaches for Notch signaling modulation: inhibition by gamma-secretase inhibitor DAPT, or by *RBPJ* knockdown and activation by overexpression of *NICD*.

It has been previously shown that Notch is extremely important for neural stem cells maintenance, differentiation and cell fate choice [5,7,15], *RBPJ*-null embryos show a delay in nervous system development, and inhibition of Notch alone is sufficient to trigger the neurogenic program in astrocytes [16]. However, according to our data Notch cascade inhibition, regardless of the used inhibition technique, has no significant effect on early stages of the differentiation of hIPS cells into DA neurons. We suppose that double SMAD inhibition, as well as activation of SHH and WNT pathways by running the neuronal differentiation program, creates a strong signaling background insensitive to Notch inhibition stimuli.

On the contrary, Notch activation had a pronounced effect on differentiation. We should emphasize that our experimental design of NICD overexpression is close to representation of supraphysiogical NICD expression in a process where Notch signaling is typically not active or is inhibited. As it has been shown previously, during differentiation of spheroids to neurons Notch, plays a role in regionalization of obtained cells, so inhibition of Notch signaling by DAPT promoted higher expression of markers characteristic of forebrain compartments [13]. Based on the data obtained in the course of our experiment, we conclude that Notch activation leads to a contrary effect. We found that activation of Notch led to downregulation of *CALB1, CAMK2A, NRGN, PIK3R3* genes, which are characteristic of pyramidal, GABA-interneurons and cholinergic neurons, at the same time, it led to the upregulation of such markers as *DAAM2, ACSBG1* typical for caudal regions, in particular, for a spinal cord.

Increased expression of *DAAM2* also leads to activation of Wnt-signaling, which is gradually activated during embryonic development with a maximum in more caudal regions of the brain and also is able to influence the Notch signaling functioning. For example, combined treatments of Wnt and Notch modulators were shown to affect brain regional identity of IPS cells-derived spheroids depending on Wnt/Notch balance [13].

In addition, in our study, the overexpression of NICD was shown to increase the expression of *CORIN*, coding for the serine protease that was originally found in the heart, but during brain development, *CORIN* is expressed in the basal plate of the neural tube, where progenitor cells of DA neurons are located. It has been shown that, in pluripotent stem cell differentiation, CORIN can serve as a marker for precursors of DA neurons, since CORIN-positive cell population contain more cells positive for TH compared with unsorted cell populations [1]. We also found an upregulation of *GDNF*, shown to promote the survival and differentiation of DA neurons in culture, and able to prevent apoptosis of motor neurons with damaged axons. These findings may also support the assumption of caudal specification induced by NICD activation.

It is known that Notch is essential for the formation of axons and dendrites in vivo [17,18] and in vitro [19]. Our data show that Notch activation is associated with downregulation of *S100B* and *TSPAN7* genes important for axon growth. S100beta protein is expressed predominantly in astrocytes, but it has been shown to play an important role in growth regulation. TSPAN7 is known to play role in regulation of cell migration [20], AMPA receptor internalization and cerebellum development, especially for cerebellar granule cell axonal branching [14].

We also found that NICD overexpression increased the expression of some types of potassium channels, *KCNA5*, as well as *KCNN3* and *KCNE4*. It was an unexpected finding because Notch signaling (through *RBPJ*) in cardiomyocytes was previously linked to the loss of active histone marks on potassium channel subunit promoters, downregulation of potassium channels [21], and attenuation of whole cell potassium currents [22,23]. Taken together, these data could indicate that Notch activation has different downstream effects in cardiomyocytes and in neurons, which confirms its context specificity [24].

NICD overexpression also led to decreased expression of *NEFM*, which codes a protein associated with neurofilaments, as well as *MAP6* and *MAP2*, which are involved in microtubule stabilization. It was shown that HES1, the downstream target of the Notch signaling, serves as a transcription repressor for *MAP2* and can directly bind to the of *MAP2* promoter [25]. Given that MAP2 is a marker of neuronal maturity, we can speculate that overexpression of NICD and Notch activation may prevent final neuronal maturation.

## 4. Materials and Methods

### 4.1. Human Induced Pluripotent Stem Cell Lines

hIPS cell lines were cultivated under standard conditions (CO_2_ 5%, O_2_ 20%, temperature 37 °C). Cells were maintained under feeder-free conditions using Geltrex (Thermo Fisher Scientific, A1413202, Paisley, UK) at a dilution of 1:100. Essential 8 Medium (Gibco, A1517001, Paisley, UK) with the addition of Penicillin-Streptomycin (Thermo Fisher Scientific) 1×. ReLeSR (STEMCELL Technologies, Catalog #05872, Vancouver, BC, Canada) was used for cell dissociation.

Three hIPS cell lines were used:

(1). hIPS cell line (AD3) was generated from HNFs using the lentiviral, nonintegrating Sendai reprogramming kit (CytoTune-iPS 2.0 Sendai Reprogramming kit (Invitrogen, Paisley, UK) according to the manufacturer’s instructions. HNFs were purchased from Lonza (Slough, UK) and were cultured as described [26]. Generated hIPS cells were cultured under feeder-free conditions and maintained on plates coated with Matrigel (growth factor reduced; BD) with mTeSR1 (STEMCELL Technologies) at 37 °C, 5% CO_2_, and 21% O_2_ according to WiCell Inc. protocols. Cells were passaged every 4–5 d at ∼80% confluence by using 0.02% EDTA (Versene). Generated AD3 Sendai-derived hIPS cell line was characterized according to protocol published before [27] and fulfilled all pluripotency criteria [26];
(2). WTSIi004-A (HPSI1113i-qolg_3) from European Bank for Induced pluripotent Stem Cells (EBiSC) Biosample ID SAMEA2464810, accessed on 10 November 2022.
(3). WTSIi046-A (HPSI0214i-wibj_2) from European Bank for Induced pluripotent Stem Cells (EBiSC) Biosamples ID SAMEA2627567, accessed on 10 November 2022.

The EBiSC Bank acknowledges the Wellcome Trust Sanger Institute (WTSI) as the source of the human induced pluripotent cell lines WTSIi046-A (HPSI0214i-wibj_2) and WTSIi004-A (HPSI1113i-qolg_3), which were generated with support from EFPIA companies and the European Union (IMI-JU’).

### 4.2. Differentiation of hIPS Cells

For differentiation of hIPS cells, a modified protocol described in Doi et al. was used [1]. hIPS cells were cultured in TeSR-E8 Basal Medium (STEMCELL) on Geltrex (Thermo)/Knockout DMEM (Gibco) 1:100. For differentiation, cells were dissociated with TrypLE Select Enzyme (Gibco) and seeded into 6 well plates coated with Geltrex/Knockout DMEM at a density of 7 × 10^5^ cells per well. During seeding, 5 μM Y27632 was added to the medium. A day after, the medium was changed to fresh, without Y27632. A day later (day 1), the medium was changed to a differentiation medium containing GMEM (Gibco), 8% Knockout serum replacement (Thermo), 0.1 mM NEAA (Gibco), 0.1 mM beta-mercaptoethanol, 1 mM Pyruvate (Gibco) and 2 mM L-Glutamine, supplemented with 500 nM A83-01 (STEMCELL), 2 μM Puromorphamine (Sigma-Aldrich, Schnelldorf, Germany), 100 ng/mL FGF8b (STEMCELL) from days 1 to 7, 100 nM LDN193189 (STEMCELL) from days 1 to 12, and 3 μM CHIR99021 from days 3 to 12. The medium was changed daily.

On differentiation day 12, cells were harvested with Accumax (STEMCELL) and plated on 24 well AggreWell-800 (STEMCELL) at a density of 1.2 million cells per well according to the manufacturer’s protocol in a medium containing Neurobasal medium (Gibco), 1% Penicillin-Streptomycin, 0.1 mM beta-mercaptoethanol, 200 mM ascorbic acid (Sigma), 2 mM L-glutamine (Gibco), 400 mM dbcAMP (STEMCELL), B-27 supplement 1x (Gibco), supplemented with 10 ng/mL GDNF (STEMCELL), and 20 ng/mL BDNF (STEMCELL) to form embryoid bodies. During seeding, 5 μM Y27632 was added to the medium. The medium was changed the next day after seeding and then every 3 days.

On day 28 of differentiation, the embryoid bodies were dissociated using Accumax (STEMCELL). The resulting cell suspension was used for final differentiation and maturation under in vitro conditions. Cells were plated on 96 well plates coated with Geltrex/Neurobasal medium (Gibco) at a density of 7.5 × 10^4^ cells per well in Neurobasal medium (Gibco), 1% Penicillin-Streptomycin, 0.1 mM beta-mercaptoethanol, 200 μM ascorbic acid (Sigma), 2 mM L-glutamine (Gibco), 400 μM dbcAMP (STEMCELL), B-27 supplement 1x (Gibco), supplemented with 10 ng/mL GDNF (STEMCELL), and 20 ng/mL BDNF (STEMCELL). During seeding, 5 μM Y27632 was added to the medium. The medium was changed the next day after seeding and then 1 time in 3 days. Differentiation was carried out up to day 53.

### 4.3. Lentiviruses Production

Lentiviral packaging plasmids were a generous gift from Didier Trono (École Polytechnique Fédérale de Lausanne, Switzerland). pLVTHM was modified as described in Kostina et al. [28] by the addition of the T7 tag and chloramphenicol resistance gene (cm), resulting in the pLVTHM-T7-cm vector. Open reading frame for murine Notch intracellular domain (NICD) was amplified from reversely transcribed mouse ES cells mRNA, using the 5’-GGCGCGCCTCTGGATCCAGTGCTGCTGTCCCGCAAG-3′ and 5’-CCACTAGTGCGGCCGCTTATTT AAATGCCTCTGGAATGTG-3′ primers. The NICD PCR fragment was cleaved with AscI and SpeI and then cloned in the frame of the T7 tag, replacing the cm gene within pLVTHM-T7-cm. Lentiviral production was performed in HEK293 cells as described previously [29]. In addition, 100-mm dishes of subconfluent 293T cells were co-transfected with 15 μg pLVTHM-T7-NICD, 5.27 μg pMD2.G and 9.73 μg pCMV-dR8.74psPAX2 packaging by the calcium phosphate method. The following day, the medium was changed to the fresh one, and the cells were incubated for 24 h to obtain high-titer virus production. Produced lentivirus was concentrated from the supernatant by ultracentrifugation, resuspended in 1% BSA/PBS and frozen in aliquots at −80 °C. The virus titer was defined by GFP-expressing virus; the efficiency of hIPS cells transduction was 80–85% by GFP. The viruses bear Notch intracellular domain (NICD) (as described previously [28]) and shRNA to *RBPJ*.

### 4.4. Efficiency Analysis of Inhibition/Activation of Notch Signaling Pathway during Differentiation of hIPS Cells

To check the efficiency of inhibiting/activation the Notch signaling, viral particles were added on the next day after cell seeding and incubated for 24 h. Medium was changed every day. Cells were taken for RNA isolation in at 96 h after transduction. Efficiency was checked by assessing the level of expression of *HEY* and *RBPJ* genes by the real-time PCR method.

### 4.5. RNA Isolation

For RNA isolation cells were grown in wells of 96 well plates. Cells were washed by PBS and isolated using ExtractRNA reagent (cat. # BC032, Evrogen, Moscow, Russian Federation). All procedures were performed according to the manufacturer’s recommendations.

### 4.6. Real-Time PCR

cDNA was synthesized from 1 μg of RNA using a RevertAid Reverse Transcriptase (cat. # EP0442, ThermoFisher Scientific). Quantitative PCRs were carried out with qPCR mix-HS SYBR (cat. # PK147L, Evrogen). The data were assessed using a delta-delta-Ct method normalized by *GAPDH*. The used primers are shown in Table 2.

### 4.7. Inhibition/Activation of Notch Signaling Pathway during Differentiation of hIPS Cells

To modulate the Notch signaling pathway, viral particles were added on the next day after cell seeding and incubated for 24 h. In addition, 10 μM DAPT (Sigma) were added to the appropriate well from days 1 to 7 of differentiation. Cells were taken for RNA isolation on day 7 of differentiation.

### 4.8. Immunofluorescent Staining

All stages of immunofluorescent staining were carried out at room temperature. The cells were fixed with 4% paraformaldehyde (Sigma) for 12.5 min. Cells were washed with PBS three times for 5 min. Permeabilization was carried out with a 0.5% Triton X-100/PBS for 5 min, followed by washing with PBS 3 times for 5 min. To block non-specific binding, samples were incubated with 5% normal horse serum diluted in PBS for 1 h. Incubation with first antibodies was carried out for 60 min, and antibodies were diluted in 5% normal horse serum in PBS at a ratio of 1:200 (list of antibodies used is given in Table 3). Secondary antibodies were diluted in 5% normal horse serum in PBS at a ratio of 1:1000. Incubation with secondary antibodies was performed for 1 h, protected from light, after which the samples were stained with an aqueous solution of DAPI at a concentration of 0.1 µg/mL.

### 4.9. Flow Cytometry

For flow cytometry, phenotyping cells were grown in wells of 96 well plates. Conditioned medium from each well was removed, and cells were dissociated with TrypLE Select Enzyme (Gibco) and dissolved in conditioned medium supplemented with 5 μM Y27632. Intracellular staining of the cells was made by Alexa Fluor^®^ 594 anti-tyrosine hydroxylase antibodies (Cat. 818003 BioLegend, London, UK) with PerFix-nc-Kit (no centrifugation assay kit) (Beckman Coulter, Marseille, France, catalog # B10825). All procedures were performed according to the manufacturer’s recommendations.

Cell phenotyping was performed using a CytoFlex S (Beckman Coulter Biotechnology, Suzhou, China) flow cytometer equipped with 405 nm (violet), 488 nm (blue), and 638 nm (red and 561 nm (yellow-green) lasers. FSC (forward scattering) and SSC (side scattering) were used as triggering signals and for primary cell detection. Threshold was set using FSC to discriminate noise and debris.

Fluorescence was measured after excitation on a yellow-green laser with 590 nm light filter. The evaluation was carried out by counting at least 5000 cells.

The flow cytometry data were analyzed using CytExpert 2.4 (Beckman Coulter, Biotechnology, Suzhou, China) and Kaluza v2.1 (Beckman Coulter, Biotechnology, Suzhou, China) software.

### 4.10. Patch Clump

Action potentials were obtained using the patch-clamp technique in the “whole-cell” configuration in the current clamp mode (I = 0) at room temperature. For the patch-clamp recordings, neurons were dissociated to a single cell suspension and seeded on coverslips covered by Geltrex (Thermo) 2–3 days before the study. Glass microelectrodes for action potentials recording were made using a puller (P-1000, Sutter Instrument, Novato, CA, USA). Action potentials were registered using an Axopatch 200B amplifier and Clampex 10.3 software (Molecular Devices Corporation, San Jose, CA, USA). The potentials were recorded at 20–50 kHz, and a 5 kHz low-pass filter was used using an ADC (Digidata 1440A acquisition system, Molecular Devices Corporation). The extracellular solution contained 140 mM NaCl, 5.4 mM KCl, 1.8 mM CaCl_2_, 1 mM MgCl_2_, 5.5 mM glucose, 10 mM HEPES, pH 7.4 (NaOH). The intracellular solution consists of 105 mM gluconate potassium, 20 mM KCl, 5 mM NaCl, 2 mM MgCl_2_, 1.5 mM MgATP, 10 mM EGTA, 10 mM HEPES and pH 7.2 (NaOH).

### 4.11. High Performance Liquid Chromatography (HPLC)

For HPLC, cell cultures were stabilized at HClO_4_ (final concentration 0.1 M) and stored at −80 °C until measurements were taken. Sample were thawed, homogenized using ultrasound, filtered in columns (PVDF, Millipore, Tullagreen, Co Cork, Ireland) by centrifugation 14,000× *g* 1 min., 4 °C before measurement. The supernatant was used for measurement of dopamine (DA), serotonin (5HT), norepinephrine (NA) and their metabolites: homovanillic acid (HVA), 3,4-dihydroxyphenylacetic acid (DOPAC) and 5-indoleacetic acid (5-HIAA) concentrations using the reverse method phase high-performance liquid chromatography with electrochemical detection. The measurement was carried out on an Eicom HTEC-500 chromatograph with WE-3G carbon electrode (Eicom, Kyoto, Japan) at +650 mV potential. Separation was carried out on a reverse phase column CA-50DS (150 × 2.1 mm, Eicom, Kyoto, Japan) with a flow rate of 200 µL/min. Composition mobile phases: per 1 L of solution: 14.9 g NaH_2_PO_4_, 1.376 g Na_2_HPO_4_, 50 mg EDTA, 400 mg sodium octylsulfonate, methanol 19.5%, pH 4.2.

### 4.12. RNA Sequencing

Quality of RNA was checked with fluorimetric analysis by Quantus and capillary electrophoresis by QIAxel. PolyA RNA was purified with Dynabeads mRNA Purification Kit (Ambion, Paisley, UK). Libraries were made from polyA RNA with NEBNext Ultra II Directional RNA Library Prep Kit for Illumina (NEB) according to the manual. Single-read sequencing was performed on Illumina HiSeq2500 with 140 bp read length, with at least 30 million reads generated for each sample.

### 4.13. Data Processing

The sequenced reads were checked for quality using the FastQC tool (http://www.bioinformatics.babraham.ac.uk/projects/fastqc/), accessed on 15 October 2022. Then, the sequenced reads were trimmed using Cutadapt v4.1 [30] with default parameters for single-strand reads. To quantify transcript expression, pre-processed reads were aligned with the reference genome with STAR [31], and gene expression was calculated with featureCounts from the Subread package [32]. Further analysis of gene expression was performed using the DeSeq2 R package [33]. Transcript variants were annotated with AnnotationDbi R package [34].

Enriched data analysis was made with the help of modEnrichr and Appyters tools [35].

### 4.14. Statistical Methods

Statistical analysis was performed using GraphPad Prism 7. At least three independent experiments (biological replicates) were performed for each measurement. All data are presented as mean ± SD.

## 5. Conclusions

The Notch signaling is essential for the development and function of the nervous system, but the effects of modulating Notch signaling are highly dependent on environment. Based on our data, we can conclude that the early stages of differentiation of hIPS cells to DA neurons are not sensitive to the inhibition of the Notch signaling cascade. At the same time, Notch activation leads to some caudal shift in differentiation, increased expression of markers, specific for DA progenitors, and a decrease of the expression of genes, which are important for the formation of axons and dendrites, as well as microtubule stabilizing proteins.

### Limitations

The purpose of this study was to investigate the effects of Notch modulation only at early stages of differentiation of hIPS cells to DA neurons. We did not evaluate the effects of this modulation on outcomes and efficiency of the differentiation because Notch inhibition by shRNA to *RBPJ* and Notch activation by overexpression of *NICD* is stable in time, and modulation of Notch signaling at the at later stages of differentiation may have very different effects. The modulation of Notch cascade at the later stages of differentiation is a subject for further studies.

## Figures and Tables

**Table 1:** Description of experimental groups.

Name of Group	Description
CONTR	Control differentiation
SH	Differentiation with inhibition of Notch signaling by shRNA to *RBPJ* (*RBPJ* knockdown)
NICD	Differentiation with activation of Notch signaling by overexpression of NICD
DAPT	Differentiation with inhibition of Notch signaling by DAPT

**Table 2:** Primers, which were used.

HEY1—F	TGAGCTGAGAAGGCTGGTAC
HEY1—R	ATCCCAAACTCCGATAGTCC
RBPJ—F	CAGTTCACAGCAGTGGGGAG
RBPJ—R	GCGGTCTGCTTATCAACTTTCC
GAPDH—F	AATGAAGGGGTCATTGATGG
GAPDH—R	AAGGTGAAGGTCGGAGTCAA

**Table 3:** Antibodies, which were used.

Antigen	Manufacturer, Source and Cat. Number
OCT4	Santa Cruz Biotechnology, Heidelberg, Germany, Oct-3/4 Antibody (N-19), sc-8628
NANOG	Sigma-Aldrich, Schnelldorf, Germany, Anti-NANOG Antibody, clone 7F7.1, MABD24
TH	Santa Cruz Biotechnology, Heidelberg, Germany, TH Antibody (H-196), sc-14007
MAP2	Sigma-Aldrich, Schnelldorf, Germany, Monoclonal Anti-MAP2 antibody produced in mouse, M4403
TH	BioLegend, London, UK, Alexa Fluor^®^ 594 anti-Tyrosine Hydroxylase, catalog# 818003

## Data Availability

The GEO (Gene Expression Omnibus) accession number for the RNA sequencing data reported in this paper is GSE218635. Accessed on 23 November 2022.

## Supplementary Materials

The following supporting information can be downloaded at: https://www.mdpi.com/article/10.3390/ijms24021429/s1.
