# Supplementary material for: Modulation of Notch Signaling at Early Stages of Differentiation of Human Induced Pluripotent Stem Cells to Dopaminergic Neurons
Source: Int J Mol Sci. 2023 Jan 11;24(2):1429. doi: 10.3390/ijms24021429 (PMC9867149; doi:10.3390/ijms24021429)
Supplement: Supplementary file 1 [file ijms-24-01429-s001.zip › ijms-2084325-supplementary.pdf]

Supplemental material.

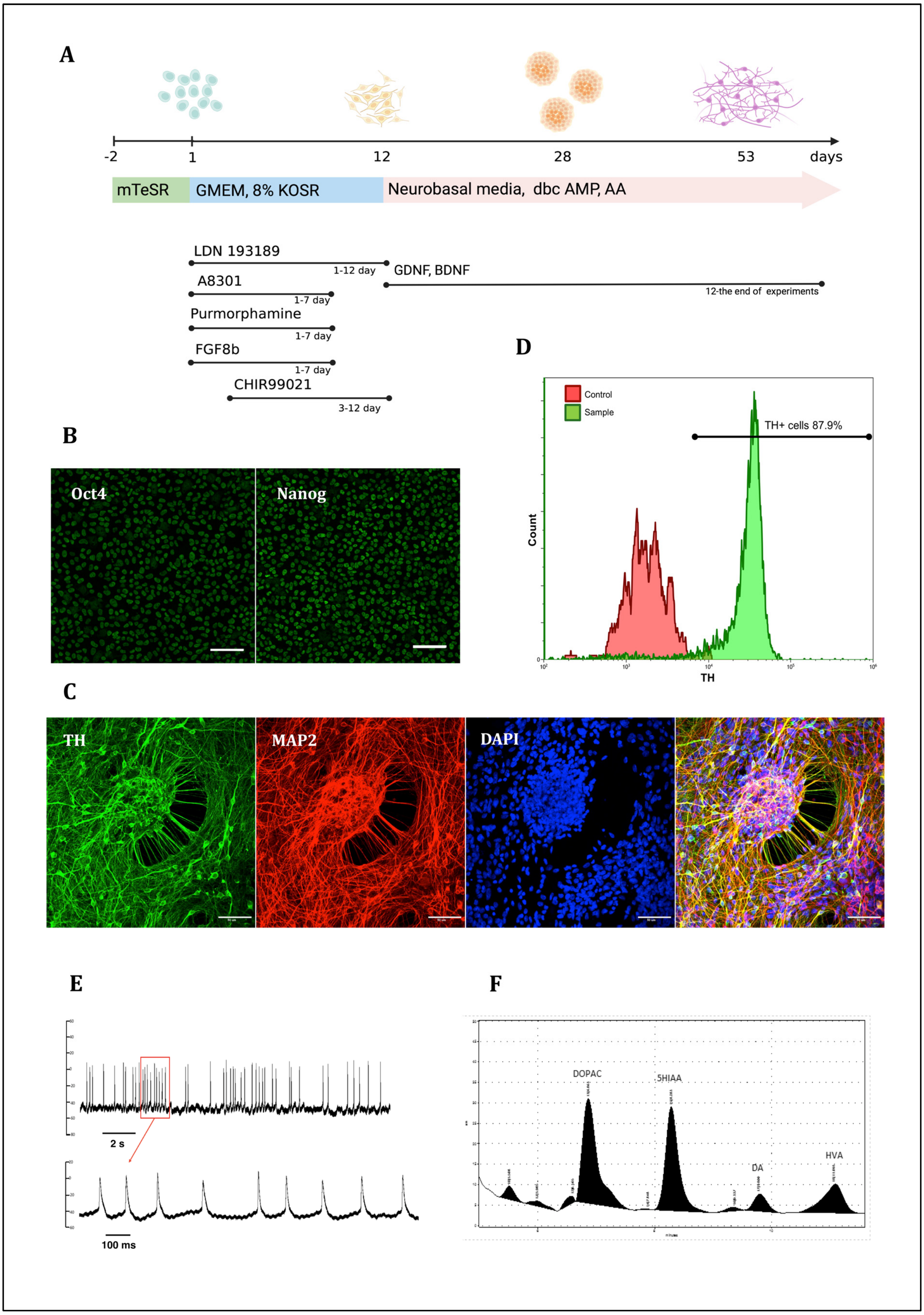

Figure S1. Differentiation of human Induces pluripotent stem (hIPS) cells to dopaminergic (DA) neurons. A. Scheme of differentiation protocol. Created in BioRender.com. B. Immunofluorescent staining of

undifferentiated hIPS cells showed staining for pluripotent markers Oct4 and Nanog. Scale bars 100 µm. C. Immunofluorescent staining of differentiated cells, day 53, showed colocalization of tyrosine hydroxylase (TH), marker of DA neurons, with MAP2, marker of mature neurons. Scale bars 50 µm. D. Flow cytometry analysis showed a large percentage of TH positive cells fraction in differentiated cells, day 53. E. Patch clamp analysis showed the ability of differentiated cells on day 53 to spontaneously generate action potentials. F. High performance liquid chromatography showed the presence of dopamine and its metabolites (DOPAC and homovanillic acid (HVA)) in obtained cells on day 53.

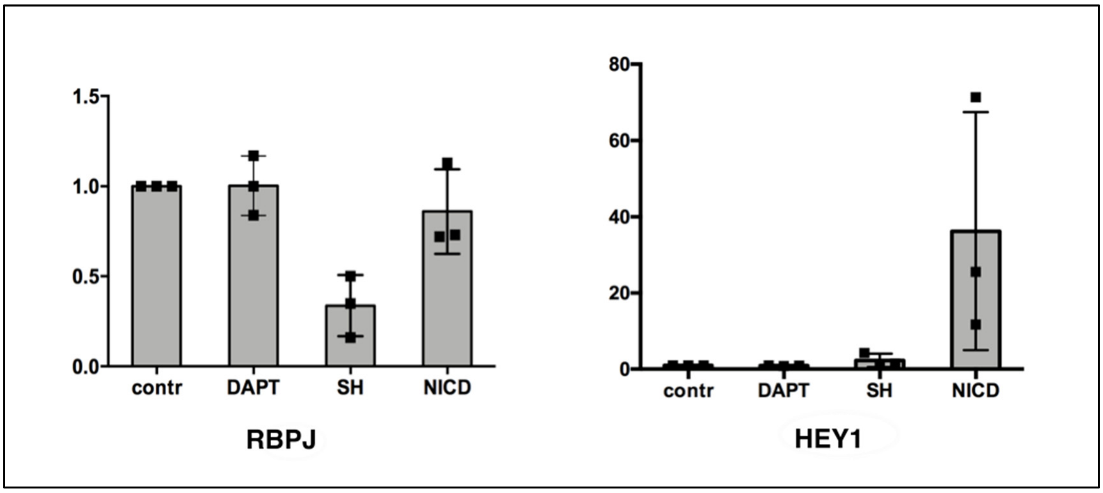

Figure S2. Real-time PCR for genes *RBPJ* and *HEY1*. Real-time PCR for genes *RBPJ* and *HEY1* during modulation of Notch signaling by shRNA to *RBPJ* and overexpression of NICD respectively showed the efficient inhibition and activation of Notch signaling respectively during differentiation of human induced pluripotent stem cells to dopaminergic neurons after 96 hours of transduction. Represented as mean ± SD.

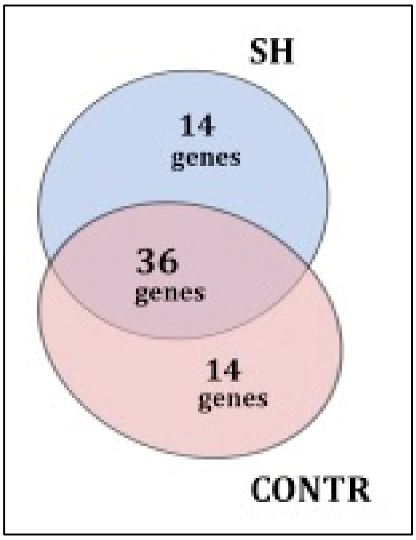

Figure S3. Comparison of gene's groups. Comparison of genes, that were obtained as overexpressed in the NICD group during comparison with SH and with CONTR groups, top 50 genes, showed dominant number of overlapping genes.

Table S1. List of genes from cluster 1, 2, 3.

Number of the Cluster	List of Genes
1	*A2ML1, AADACL3, ABCB1, ABCC2, ABCD1, ABCF1, ABCF2, ABCG1, ABHD12B, ABHD17C, ABLIM2, ABRACL, ABT1, ACAD8, ACAP1, ACAT2, ACBD6///LHX4-AS1, ACE, ACOT9, ACPP, ACSL1, ACSL6, ACSM6, ACTB, ACTC1, ACTL6A, ACTL6B, ACTR5, ACTR8, ACTRT3, ACVR1C, ADAM20P1, ADAM28, ADAM8, ADAMTS16, ADAMTS19, ADAMTS5, ADAMTS8, ADAP1, ADARB2, ADCY1, ADCY2, ADCY5, ADGRE2, ADGRG2, ADIPOR1, ADM2, ADNP2, AEBP2, AEN, AFP, AGMAT, AGO1, AGO2, AHR, AHSA1, AHSG, AIF1, AIFM2, AIMP2, AIPL1, AK5, AK6, AKAP1, AKR1C3, ALDH18A1, ALDH1A1, ALDH1L1, ALG1L2, ALG3, ALG6, ALKAL2, ALKBH2, ALS2CL, ALYREF, AMD1, AMER1, ANAPC1, ANAPC5, ANK1, ANK3, ANKEF1, ANKH, ANKLE1, ANKRD27, ANKRD29, ANKRD34B, ANKS6, ANO4, ANO9, ANOS1, ANP32E, ANXA11, ANXA3, AOX1, AP1M2, AP1S3, APBB2, APELA, APH1B, APIP, APPL1, AQP3, AQP4-AS1, AQP5, ARFGEF1, ARHGAP1, ARHGAP12, ARHGAP22, ARHGAP23, ARHGAP31, ARHGAP8, ARHGEF16, ARHGEF5, ARID5A, ARIH2, ARL11, ARMC6, ARMT1, ARPC5, ARPC5L, ARRB1, ASB12, ASB6, ASF1B, ASH2L, ATAD3A, ATAD3B, ATF1, ATG2B, ATG3, ATG5, ATL3, ATP1A3, ATP23, ATP6V0A2, ATP6V1E2, ATP8A1, ATP8B1, ATR, AUH, AUNIP, AUP1, B3GAT1, B3GAT2, B3GNT2, B3GNT3, B3GNT4, B3GNT7, BAMBI, BANK1, BARD1, BATF3, BBC3, BCAR1, BCL11A, BCL11B, BCL2L10, BDH1, BEND3, BEST2, BFAR, BHLHA15, BHMT, BICD1, BICDL2, BID, BIK, BLVRA, BMPR1A, BMS1, BNC2, BNIPL, BOLA3, BOP1, BRAP, BRD9, BRI3BP, BRINP1, BRMS1, BSPRY, BTBD11, BUB1B, BUD23, BUD31, BYSL, C10orf82, C11orf24, C12orf43, C12orf56, C12orf66, C12orf75, C14orf119, C15orf39, C16orf54, C16orf74, C16orf91, C17orf53, C17orf58, C17orf75, C18orf21, C19orf73, C1orf112, C1orf116, C1orf127, C1orf210, C1orf216, C1orf226, C1orf61, C1orf94, C1QBP, C1QL3, C1QTNF2, C2CD2, C2orf50, C2orf80, C3orf52, C4orf19, C4orf51, C5orf47, C6orf136, C6orf223, C7orf43, C7orf57, C9orf116, C9orf135, C9orf78, CA12, CA2, CA5BP1, CA7, CABYR, CACNA2D1, CACNA2D3, CACYBP, CALCR, CALN1, CAMK2N2, CAMK4, CAMKV, CAPG, CAPN13, CAPNS1, CARD10, CARD11, CARMIL1, CARMIL3, CARS, CASP3, CASZ1, CBLN1, CBR1, CBS///CBSL, CBX7, CCDC137, CCDC17, CCDC172, CCDC186, CCDC28A, CCDC43, CCDC63, CCDC71, CCDC86, CCKBR, CCL26, CCL28, CCNA2, CCNB1, CCNC, CCNE1, CCNF, CCNI2, CCNQ, CCNYL1, CCT2, CCT5, CCT6A, CD177, CD19, CD1D, CD200, CD247, CD2AP, CD34, CD3EAP, CD8B, CDC123, CDC16, CDC25A, CDC26, CDC37L1, CDC40, CDC45, CDC6, CDCA4, CDCA5, CDCA7L, CDCP1, CDH26, CDHR1, CDHR3, CDK14, CDK15, CDK2AP1, CDK5, CDK7, CDK9, CDR2L, CDS1, CEACAM1, CEBPA, CEBPB, CEBPZ, CEBPZOS, CENPM, CENPP, CENPU, CENPW, CEP72, CER1, CGB2, CGN, CGNL1, CHAC1, CHAC2, CHAF1A, CHAF1B, CHCHD3, CHCHD4, CHCHD7, CHDH, CHEK2, CHGA, CHKA, CHML, CHMP1B, CHMP4C, CHN1, CHODL, CHP2, CHRAC1, CHRNB2, CHRNB4, CHST2, CHST4, CHST8, CHST9, CHSY1, CKMT1A, CKMT1B, CKMT2, CLCA4-AS1, CLDN1, CLDN10, CLDN6, CLDN7, CLDN9, CLINT1, CLN6, CLPTM1L, CLTA, CMAS, CMSS1, CMYA5, CNDP2, CNGA1, CNIH4, CNMD, CNN1, CNN2, CNOT11, CNST, CNTLN, CNTN1, COA3, COA4, COA6, COA7, COBL, COCH, COG2, COL28A1, COL8A2, COMMD10, COMMD4, COMMD5, COMMD7, COMTD1, COPS3, COPS5, COQ3, COQ9, CORO2A, COX16, COX17, COX6B1, CP, CPLX1, CPNE7, CPO, CPPED1, CPT1A, CR1L, CR2, CRB3, CREB3L4, CREG2, CRIP3, CRIPT, CRLF3, CRNKL1, CRTC2, CRYAB, CRYBB3, CRYBG1, CRYGD, CRYGN, CRYM, CRYZL2P, CSE1L, CSTB, CSTF2, CTBP2, CTH, CTR9, CTSC, CTU2, CUL4A, CUX2, CWF19L1, CXCL3, CXCR2, CXorf56, CYB561, CYB5A, CYCS, CYP27B1, CYP2A13, CYP2F1, CYP2J2, CYP2S1, CYP4X1, DAB1, DACT2, DAPK1, DARS2, DAXX, DCAF13, DCAF4, DCLK1, DCP2,*

	DCTPP1, DCUN1D5, DDAH1, DDIAS, DDIT4L, DDR2, DDX11, DDX25, DDX28, DDX39A, DDX3X, DDX46, DDX51, DDX55, DEC1, DENND1C, DENND2C, DEPDC1, DEPDC1B, DEPDC7, DERL3, DESI2, DEXI, DFFB, DGKE, DGKZ, DHCR24, DHDDS, DHRS3, DHX15, DHX33, DHX34, DHX9, DIAPH3, DIDO1, DIRAS1, DIS3L, DISP2, DLGAP3, DLL3, DMKN, DNAAF2, DNAJA2, DNAJB6, DNAJC30, DNER, DNTTIP1, DOCK5, DPH2, DPH3, DPP4, DPPA2, DPPA3, DPY30, DPYS, DPYSL3, DSCC1, DSE, DSN1, DTD2, DTWD2, DTYMK, DUS3L, DUSP23, DUSP26, DUSP5, DUSP6, DUT, DYNLL1, E2F1, E2F4, EAF2, EBNA1BP2, ECE2///EEF1AKMT4, ECEL1P2, ECT2, EDARADD, EDIL3, EEF1AKNMT, EEF1E1, EEF2KMT, EFHD2, EFR3A, EGFL6, EHBP1, EHMT1, EIF2B1, EIF2S1, EIF2S2, EIF3K, EIF3M, EIF4E3, EIF4EBP1, EIF4G1, EIF5A, ELAC2, ELMO3, ELOVL7, EMC8, EMC9, EMG1, EMX1, ENTPD1, ENTR1, EOGT, EPB41L4A, EPCAM, EPHA1, EPHA10, EPHB6, EPHX3, EPHX4, EPPK1, EPS8L1, ERCC6L, ERICH1, ERICH5, ERRFI1, ERVMER34-1, ESRG, ESRP1, ESRP2, ESYT3, ETNPPL, ETS2, ETV1, ETV5, ETV6, EWSR1, EXO1, EXOSC3, EXOSC4, EXOSC9, FA2H, FAAH, FABP5, FABP6, FAM102A, FAM102B, FAM110A, FAM110C, FAM120A, FAM124A, FAM124B, FAM129A, FAM155B, FAM174B, FAM181B, FAM19A4, FAM20A, FAM20C, FAM241A, FAM50B, FAM78B, FAM83B, FAM83D, FAM83F, FAM84A, FAM86EP, FARSB, FASTKD2, FASTKD3, FBLL1, FBXL16, FBXL21, FBXL5, FBXO25, FBXO27, FCF1, FCGR2A///FCGR2C, FCN3, FDFT1, FEN1, FERMT1, FEZF1, FGD2, FGD3, FGF13, FGF13-AS1, FGF2, FHL2, FKBP4, FLAD1, FLJ42393, FLJ45513, FLOT2, FLVCR1, FMO1, FOXD3, FOXI2, FOXL2, FOXL2NB, FOXM1, FOXN3, FOXO1, FRAT1, FRAT2, FRMD5, FTSJ1, FUS, FUT2, FXN, FXYD7, FZD5, G3BP1, GABPB1, GABRA5, GABRB3, GABRE, GABRQ, GAD2, GAL, GALM, GALNT13, GALNT14, GALNT3, GALNT6, GALNT7, GALR1, GALR3, GAREM2, GARS, GART, GATC, GATM, GCAT, GCH1, GCLM, GCNT4, GDAP1L1, GDF3, GEMIN4, GEMIN6, GEMIN7, GFOD1, GFPT2, GHRLOS, GINS4, GIPC2, GJA4, GJA5, GJB7, GLA, GLB1L3, GLDC, GLIPR1L1, GLS, GLS2, GLTP, GLYR1, GMNN, GNA12, GNA14, GNG12, GNG4, GNGT2, GNL2, GNL3, GNL3L, GNMT///CNPY3-GNMT, GNPNAT1, GNPTAB, GOLPH3, GOLPH3L, GOLT1A, GPANK1, GPAT3, GPATCH4, GPC4, GPD1L, GPD2, GPLD1, GPNMB, GPR158, GPR160, GPR176, GPR27, GPR3, GPR37, GPR39, GPR62, GPR63, GPR83, GPRC5A, GPRIN1, GPRIN3, GRAMD2B, GRB7, GRHL2, GRIA4, GRID2, GRIK4, GRIN1, GRM1, GRM2, GRPEL1, GRPEL2, GRPR, GRTP1, GRWD1, GSG1, GSKIP, GSR, GTPBP4, GUCD1, GUCY2C, GULP1, HACL1, HAPLN3, HAS3, HASPIN, HAUS8, HCCS, HCLS1, HCN1, HCN2, HCN4, HEATR5A, HECW1, HELB, HENMT1, HEPHL1, HERC5, HESX1, HGH1, HHEX, HIRA, HIST1H2AE, HIST1H2BI, HIST1H3F, HIST1H4D, HIST1H4J, HIST2H2AC, HLA-DPB1, HLA-F, HLF, HMGA1, HMGB3, HMGN5, HMOX2, HMSD, HNF4G, HNRNPAB, HNRNPD, HNRNPM, HNRNPU, HOMER2, HOOK2, HOXB13, HPGD, HPRT1, HPS6, HRASLS5, HS3ST3A1, HSD11B2, HSPA12B, HSPA14, HSPA9, HTATIP2, HTR1B, HTR6, HTR7, HUS1, HYLS1, ICA1, ICAM3, ID1, IDH1, IDO1, IER2, IFNLR1, IGFLR1, IGSF21, IGSF9B, IKBKE, IKZF5, IL15, IL17C, IL17RD, IL20RB, IL21R, IL23A, IL27RA, IL4R, IL6R, ILDR1, ILF3, ILKAP, IMP4, INAFM2, INHBA, INKA1, INO80D, INPP5A, INSR, INTS13, INTS5, IPO11///IPO11-LRRC70, IPO5, IPW, IRAK2, IRAK3, IRF2BPL, IRF5, IRX4, ISG20L2, ITGA2, ITGAM, ITGB1BP2, ITGB5, ITIH5, ITLN2, ITM2A, ITPA, ITPK1, ITPR2, ITPRIPL1, JADE1, JARID2, JAZF1, JDP2, JMY, JPH1, JPT1, JTB, KCMF1, KCNB2, KCNC4, KCND2, KCNE3, KCNG3, KCNH6, KCNJ6, KCNK1, KCNK6, KCNMA1, KCNMB1, KCNMB2-AS1, KCNMB4, KCNN2, KCNQ3, KCNS1, KCNS3, KCNV1, KCTD14, KCTD8, KDM4A, KDR, KEL, KIAA0040, KIF11, KIF14, KIF18B, KIF1BP, KIF1C, KIF23, KIF5B, KLB, KLC3, KLF15, KLHL34, KLHL7, KLK1, KLK13, KLK14, KLKB1, KLRG1, KLRG2, KNOP1, KRT18, KRTCAP3, KTI12, KYNU, L1TD1, L3MBTL2, LAD1, LAMA3, LAMC2, LAMC3, LARGE2, LARP1B, LARS, LCK, LCP1, LDLRAD4, LDLRAP1, LEAP2, LEFTY1, LEFTY2,

	LEMD3, LETM1, LHFPL2, LHFPL4, LIMD1, LIMD2, LIMS2, LINC-ROR, LINC00337, LINC00449, LINC00562, LINC00605, LINC00652, LINC00678, LINC00852, LINC00882, LINC00899, LINC00937, LINC01018, LINC01108, LINC01311, LINC01359, LINC01484, LINC01487, LINC01829, LINC01844, LINC02036, LINC02166, LINC02188, LINC02362, LINC02365, LINC02436, LINGO2, LINP1, LLGL2, LMO7, LNX1, LOC100128770, LOC100130476, LOC100288748, LOC100631378, LOC101927551, LOC101928123, LOC101928254, LOC102724159, LOC102724200, LOC102724859, LOC105371730, LOC105373884, LOC105374546, LOC105374952, LOC105375431, LOC105378421, LOC283387, LOC283731, LOC286059, LOC389641, LOC441086, LOC441666, LOC645752, LOC728975, LOC729683, LPAR3, LPCAT1, LPL, LPO, LRAT, LRP8, LRR1, LRRC1, LRRC15, LRRC3B, LRRC45, LRRC47, LRRC69, LRRFIP1, LRRK1, LRRN4, LRWD1, LSG1, LSM10, LSM3, LTF, LTO1, LTV1, LUZP2, LY75, LYAR, LYPD3, LYPD6, LYPD6B, LYPLA2, LYSMD2, LZTS1, MACC1, MACC1-AS1, MAD2L1BP, MAEA, MAF1, MAFA, MAFF, MAGEB17, MAGI2, MAGOH, MAK16, MAL2, MALSU1, MAMDC2, MANF, MAP3K21, MAP3K5, MAP4K3, MAPK13, MAPK9, MAPKAP1, MARC2, MARCH3, MARCH4, MARK1, MARS, MARS2, MASTL, MAT1A, MATK, MATN3, MB21D2, MBD2, MBOAT1, MBP, MCF2, MCM10, MCM4, MCM5, MCM6, MCM7, MCMBP, MCMDC2, MCOLN2, MDH1, MDN1, MED14OS, MED20, MED21, MED27, MEDAG, MEGF10, MELK, MET, METAP1, METTL1, METTL21A, METTL2A, METTL3, METTL5, METTL7A, MFAP3L, MFSD13A, MFSD2A, MFSD5, MFSD6, MFSD6L, MGME1, MGST1, MINDY2, MIOS, MIR17HG, MIR2052HG, MIR302C, MIR302D, MIR3671, MIR3945, MIR4455, MIS18A, MIXL1, MKLN1, MKLN1-AS, MLKL, MLYCD, MMACHC, MMP24, MNAT1, MOB4, MOCOS, MOCS3, MORC2, MOSPD1, MPL, MPP1, MPP6, MPV17L2, MPZL2, MPZL3, MREG, MRI1, MRM1, MRM3, MRO, MRPL13, MRPL15, MRPL16, MRPL17, MRPL18, MRPL19, MRPL20, MRPL24, MRPL3, MRPL32, MRPL34, MRPL35, MRPL36, MRPL39, MRPL42, MRPL44, MRPL47, MRPL48, MRPL49, MRPL51, MRPL52, MRPL55, MRPL58, MRPS10, MRPS11, MRPS12, MRPS15, MRPS16, MRPS18A, MRPS18C, MRPS21, MRPS23, MRPS28, MRPS30, MRPS34, MRPS35, MRPS36, MRPS9, MRRF, MRS2, MRTO4, MSC-AS1, MSH2, MSTO1, MT1F, MT1G, MT1H, MT1X, MTA3, MTAP, MTFR2, MTG1, MTHFD1, MTHFD1L, MTHFD2, MTHFS, MTMR12, MTMR8, MTPAP, MTX2, MUC3A, MUC4, MUC5B, MYBBP1A, MYBL2, MYC, MYCN, MYH2, MYL6, MYL7, MYLIP, MYLPF, MYO18B, MYO1D, MYO1E, MYO3A, MYOZ3, MYRFL, MYRIP, MYSM1, MYZAP, MZB1, N4BP1, N4BP2L1, N4BP3, NAA15, NAA20, NAA25, NAAA, NAF1, NAGK, NANOG, NANOGP8, NANS, NAP1L3, NASP, NAT2, NAV3, NBEAL2, NCAPG2, NCAPH, NCAPH2, NCEH1, NCLN, NDUFA7, NDUFAF4, NDUFAF5, NDUFAF7, NDUFB9, NDUFS3, NDUFS6, NDUFV3, NECAB1, NECTIN1, NEFH, NEK3, NEK8, NET1, NETO1, NF2, NFE2L3, NFIA, NFKBIB, NGDN, NHP2, NIFK, NIP7, NIPAL1, NIPAL3, NKAIN1, NKILA, NKX1-2, NLGN4X, NLN, NLRP12, NLRP2, NME1, NMI, NMNAT2, NMNAT3, NOC2L, NOC3L, NOC4L, NOCT, NOD2, NODAL, NOL11, NOL6, NOL7, NOM1, NOP10, NOP14, NOP56, NOP9, NOS1AP, NOS2, NPAP1, NPIPA1///LOC102724993, NPLOC4, NPM2, NPY2R, NR1I3, NR2E3, NR5A2, NRADDP, NRBP1, NRK, NSUN2, NSUN7, NT5DC3, NTMT1, NTS, NTSR1, NUDCD1, NUDT15, NUDT9, NUFIP1, NUP153, NUP155, NUP35, NUP37, NUP85, NUP88, NUS1, NUTF2, NUTM1, OAT, OCIAD2, OGFOD1, OGFRP1, OIP5, OLFM1, OMA1, ONECUT1, ORC1, ORC2, ORMDL2, OSBP2, OTUD6B, OTULINL, OVOL1, OVOL2, OXCT2, OXNAD1, P2RX5, P2RX5-TAX1BP3, P2RY1, PAH, PAIP2, PAK6///BUB1B-PAK6, PALM3, PAPOLG, PAPSS2, PAQR4, PARD6A, PARP12, PARPBP, PAWR, PAXIP1, PCBD2, PCDH1, PCDH12, PCDHGA6, PCID2, PCK2, PCP4L1, PCYT2, PDCD11, PDCD2, PDCD2L, PDCL3, PDE12, PDE4B, PDE5A, PDE6G, PDLIM5, PDSS1, PDZD8, PEBP1, PEX1, PEX5L, PFAS, PFDN6, PGAM5, PGM2, PGM2L1, PGRMC1, PHAX, PHC1, PHETA2, PHF5A, PHGDH, PHLPP1,

	PHOSPHO1, PI4K2B, PIF1, PIK3AP1, PIK3CB, PIM2, PIN4, PINX1, PIP4K2A, PIP5KL1, PIWIL2, PKHD1L1, PKIA-AS1, PKMYT1, PKNOX1, PKP1, PKP3, PLA2G4C, PLA2G7, PLA2R1, PLAC9, PLAUR, PLBD1, PLD5, PLEKHD1, PLEKHF1, PLK4, PLPP1, PLPP2, PLPP6, PLS1, PLSCR1, PLSCR4, PMAIP1, PMEPA1, PNMT, PNO1, PNP, PNPLA3, POC1A, POLA1, POLE, POLE2, POLR1B, POLR2F, POLR2L, POLR3E, POLR3G, POLR3K, POMP, PON1, POP1, POP4, POP5, POP7, POU2F3, POU5F1P3, POU5F1P4, PPARGC1B, PPIF, PPIG, PPIL1, PPM1A, PPM1B, PPM1G, PPM1H, PPP1R14B, PPP1R17, PPP1R1B, PPP1R7, PPP2R1B, PPP2R2C, PPP3CA, PPP3R1, PPP6R3, PPRC1, PPTC7, PRCC, PRDM14, PRDX1, PRDX6, PRELID1, PREX2, PRIM1, PRIM2, PRIMPOL, PRKAR1B, PRKAR2B, PRKCI, PRKD3, PRLR, PRMT3, PROCR, PROK2, PROKR1, PROKR2, PROM1, PROM2, PRPF31, PRPF4, PRPS2, PRR13, PRR15, PRRG2, PRRG4, PRSS16, PRSS8, PRTFDC1, PSAT1, PSIP1, PSMA3, PSMB3, PSMB4, PSMC1, PSMC2, PSMC4, PSMC6, PSMD1, PSMD11, PSMD12, PSMD14, PSMD6, PSMD7, PSMG1, PSMG4, PSPH, PTAFR, PTBP3, PTCHD1, PTGES3, PTMA, PTPN5, PTPRB, PTPRG, PTPRZ1, PTRH2, PUM3, PUS1, PUS7, PUSL1, PWP1, PWWP2A, PYCARD, QRSL1, QSER1, QTRT2, RAB15, RAB17, RAB19, RAB25, RAB31, RAB5A, RABEPK, RAD1, RAD18, RAD51, RAD54L, RALA, RAMAC, RAMP1, RAMP3, RANBP1, RANGAP1, RAPGEF3, RARRES2, RARRES3, RARS, RASAL2-AS1, RASAL3, RASD1, RASEF, RASGEF1A, RASGRF2, RASGRP2, RASL11A, RASSF10, RASSF3, RASSF9, RBBP5, RBBP8NL, RBM14, RBM19, RBM28, RBM39, RBM47, RBMXL1, RBP7, RBPMS-AS1, RBPMS2, RCC2, RDH11, RDH14, REEP5, RELL2, REPS2, REXO2, RFC2, RFC3, RFLNA///ZNF664-RFLNA, RFWD3, RGL3, RGS17, RGS5, RHBDL3, RHEBL1, RHOD, RHOH, RIF1, RILP, RIMKLB, RIMS4, RINT1, RIOK1, RIOX1, RIPOR2, RMC1, RMI2, RMND5B, RNASEH1, RNASEH2A, RNASEH2B, RNF114, RNF125, RNF13, RNF144B, RNF145, RNF175, RNF182, RNF25, RNF44, ROBO3, RPARP-AS1, RPF2, RPIA, RPL22L1, RPL36AL, RPL3L, RPP25, RPP30, RPP40, RPRM, RPS26, RPS6KL1, RPUSD1, RPUSD2, RRAS2, RRP1, RRP12, RRP15, RRP1B, RRP36, RRP9, RRS1, RSU1, RTCA, RTN4IP1, RTP1, RUBCNL, RUNDC3A, RUNX1T1, RUVBL1, RYR3, S100A14, SAAL1, SACM1L, SAE1, SAMD3, SAP18, SAP30L, SAPCD2, SAV1, SBSPON, SCARF2, SCG3, SCIN, SCLY, SCN8A, SCNM1///TNFAIP8L2-SCNM1, SCNN1A, SCNN1G, SCO1, SDC4, SDHB, SDHD, SEC14L4, SEC1P, SEH1L, SEL1L3, SELENOS, SEMA3A, SEMA6A, SEMA6A-AS1, SEPHS2, SERPINE2, SETD3, SETD4, SETD6, SF3B4, SFN, SFT2D1, SFTPD, SFXN2, SGK3///C8orf44-SGK3, SGMS1, SGO1, SH2B3, SH2D3A, SH2D4A, SH3BP5L, SH3GL2, SH3GL3, SH3YL1, SHCBP1, SHE, SHISA9, SHLD2, SHPK, SHQ1, SIRT1, SIRT7, SIVA1, SIX2, SIX4, SIX6, SKA3, SKIL, SLA, SLC10A4, SLC13A3, SLC15A1, SLC16A12, SLC19A2, SLC19A3, SLC20A1, SLC22A1, SLC22A20P, SLC22A3, SLC25A12, SLC25A17, SLC25A19, SLC25A28, SLC25A38, SLC25A4, SLC26A7, SLC26A8, SLC27A2, SLC27A4, SLC27A6, SLC29A3, SLC30A2, SLC30A3, SLC35B1, SLC35D3, SLC35F3, SLC37A1, SLC38A1, SLC38A2, SLC39A14, SLC43A3, SLC44A3, SLC45A1, SLC46A1, SLC4A4, SLC51A, SLC52A1, SLC5A6, SLC7A14, SLC7A2, SLC7A3, SLC7A6, SLCO3A1, SLCO4A1, SLCO4C1, SLCO5A1, SLED1, SLFN13, SLIRP, SLITRK3, SLMAP, SMARCA2, SMC1B, SMC4, SMCO4, SMIM10, SMIM10L2B, SMIM12, SMIM13, SMIM4, SMPDL3B, SMS, SMYD5, SNAI3, SNAP23, SNAPC5, SNHG4, SNN, SNORA3B, SNRNP25, SNRPC, SNUPN, SNURF, SNX22, SNX24, SOCS2, SOCS2-AS1, SOCS3, SOD1, SORT1, SOWAHC, SOWAHD, SOX15, SOX4, SP100, SP140, SP4, SPAAR, SPAG5, SPATA2, SPATA24, SPATS2L, SPHK2, SPIB, SPIN1, SPR, SPRY1, SPRY4, SPRYD4, SPTB, SPTBN5, SRF, SRFBP1, SRM, SRP19, SRPK1, SRPRB, SRRT, SRSF2, SRSF3, SRSF7, SS18L2, SSBP1, SSSCA1, SSTR1, SSTR2, ST14, ST6GAL1, ST7///ST7-OT3, ST8SIA5, STAB1, STAC3, STAMBPL1, STEAP1, STEAP1B///LOC401312, STEAP2, STEAP3, STK35, STMN1, STMN3, STOM, STRN, STYK1, SUCLA2, SUDS3, SUPV3L1, SURF2, SURF6, SUV39H1, SVOPL, SYK, SYN3, SYNCRIP, SYNE4, SYNJ2, SYNPO2, SYNPR, SYPL1,

	SYPL2, SYT1, SYT12, SYT2, SYT3, SYT6, TAC1, TACR3, TACSTD2, TAF4B, TAF5L, TANGO6, TAOK3, TARS, TASP1, TATDN2, TBC1D2, TBC1D22B, TBC1D4, TBC1D8B, TBCC, TBRG1, TC2N, TCAP, TCF24, TCP1, TDGF1, TDP1, TDP2, TDRD12, TDRD5, TEAD4, TEC, TEDC2, TEKT3, TELO2, TENT5B, TERF1, TES, TESC, TEX10, TEX13B, TEX41, TFAP2A-AS2, TFAP4, TFB2M, TFCP2L1, TFPI2, TGFA, THAP1, THAP11, THBS1, THOC6, THOC7, THY1, TICRR, TIGAR, TIMM10, TIMM17A, TIMM22, TIMM23, TIMM44, TIMM8A, TIMM8B, TIMMDC1, TINAGL1, TIPIN, TK1, TLCD1, TLE1, TLL2, TLR2, TLR3, TM2D2, TM4SF18, TMA16, TMC5, TMEFF2, TMEM104, TMEM11, TMEM110-MUSTN1, TMEM120B, TMEM125, TMEM139, TMEM145, TMEM151A, TMEM151B, TMEM165, TMEM177, TMEM178A, TMEM183A, TMEM200A, TMEM201, TMEM203, TMEM220, TMEM220-AS1, TMEM223, TMEM238, TMEM266, TMEM268, TMEM30B, TMEM33, TMEM37, TMEM38A, TMEM52, TMEM59L, TMEM63A, TMEM69, TMEM82, TMEM86A, TMEM86B, TMEM87A, TMEM8A, TMIE, TMPPE, TMPRSS13, TMPRSS4, TMPRSS6, TMTC1, TMTC3, TMX2, TNFRSF10A, TNFRSF11A, TNFRSF12A, TNFRSF8, TNFSF11, TNIK, TNIP2, TNMD, TNNC2, TOMM22, TOMM40, TOP1MT, TOR3A, TOX, TP53, TP73, TPM3, TPRN, TRAF3IP2, TRAIP, TRAPPC13, TRAPPC8, TRDN, TRHDE-AS1, TRIAP1, TRIM14, TRIM24, TRIM35, TRIM54, TRIM6, TRIM65, TRIM72, TRIP13, TRMT10C, TRMT12, TRMT2B, TRMT44, TRMT5, TRMT61A, TRMT61B, TRNP1, TROAP, TRPA1, TRPM6, TRUB2, TSHZ3, TSKU, TSLP, TSN, TSR1, TSTA3, TTC19, TTC27, TTC39B, TTC39C, TTC9, TTI2, TTK, TTLL12, TUBB6, TUFM, TULP1, TULP2, TUSC2, TWISTNB, TWNK, TXLNG, UBALD1, UBB, UBE2E1, UBE2F, UBE2J2, UBFD1, UBIAD1, UBQLN1, UBTF, UCHL1, UCHL3, UCK2, UCKL1, UCMA, UFD1, UGP2, UGT8, UHRF1BP1L, ULK1, UMPS, UNC5D, UNG, UPRT, UQCC3, UQCRQ, URB2, URGCP, URI1, USH1G, USH2A, USP12, USP18, USP2-AS1, USP28, USP32, USP43, USP44, USP48, UTF1, UTP11, UTP14A, UTP15, UTP20, UTP25, UTP3, UTP4, UTP6, VANGL1, VASP, VAV1, VCAN, VCL, VDR, VENTX, VIL1, VIPR1, VIPR2, VKORC1L1, VOPP1, VPREB3, VPS33A, VPS9D1-AS1, VSIG10///LOC101928274, VSNL1, VSTM5, WARS, WASHC5, WASL, WDR1, WDR3, WDR4, WDR43, WDR44, WDR45, WDR46, WDR72, WDR77, WDR82, WSB2, WWC1, WWC2, XG, XK, XKRX, YARS2, YJU2, YRDC, YTHDC2, YTHDF1, YWHAB, ZAN, ZAR1L, ZBTB11-AS1, ZBTB2, ZBTB3, ZBTB44, ZC3H4, ZCCHC17, ZCCHC2, ZDHHC13, ZDHHC16, ZDHHC19, ZDHHC2, ZDHHC20, ZDHHC22, ZDHHC5, ZFAND6, ZFP42, ZFYVE28, ZMAT4, ZMYND19, ZNF101, ZNF114, ZNF121, ZNF134, ZNF142, ZNF202, ZNF212, ZNF281, ZNF35, ZNF385B, ZNF385D, ZNF394, ZNF398, ZNF483, ZNF485, ZNF488, ZNF512B, ZNF525, ZNF564, ZNF572, ZNF587B, ZNF613, ZNF616, ZNF622, ZNF630, ZNF648, ZNF649, ZNF684, ZNF697, ZNF732, ZNF749, ZNF765, ZNF770, ZNF813, ZNF827, ZNF845, ZNF850, ZNF860, ZNF878, ZNF90, ZNHIT2, ZPR1, ZSCAN10, ZSCAN2, ZSWIM3, ZUP1, ZWILCH, ZXDC, ZYG11A
2	*A2M, A2M-AS1, AATK, ABAT, ABCA1, ABCA13, ABCA17P, ABCA3, ABCA5, ABHD10, ABHD15, ABHD6, ABRAXAS1, ACADVL, ACBD5, ACCS, ACOXL-AS1, ACP2, ACRBP, ACSS1, ACSS3, ACTA2, ACTN3, ACVR1B, ADAL, ADAM17, ADAMTS13, ADAMTS6, ADAMTS9, ADAMTSL3, ADCY7, ADD3, ADGRG1, ADGRV1, ADNP, ADORA2A, ADORA2A-AS1, ADPRM, ADSSL1, AFF4, AGBL5, AGFG2, AGO3, AGPAT3, AGPAT4, AHDC1, AHNAK, AHSA2P, AJUBA, AK7, AKAP13, AKAP6, AKIP1, ALAD, ALDH1A2, ALDH5A1, ALG9, ALOX12B, AMN1, AMOTL1, AMPH, ANAPC16, ANGPTL4, ANKMY2, ANKRD13A, ANKRD34A, ANKRD42, ANKRD53, ANO3, ANP32A, ANP32B, AP1M1, AP3M1, AP3M2, APC2, APLP1, APLP2, APMAP, APOBEC3H, APOLD1, APP, ARFGAP3, ARHGAP10, ARHGAP15, ARHGAP24, ARHGAP29, ARHGAP6, ARHGEF17, ARHGEF25, ARHGEF37, ARHGEF4, ARHGEF9, ARID1B, ARID2, ARID3A, ARIH2OS, ARL10, ARL3, ARL4A, ARL4C, ARL5B, ARL6IP1, ARL6IP5, ARMC1, ARMC12, ARMC3,*

	ARMCX1, ARMCX3, ARMCX6, ARNT, ARRDC3, ARRDC4, ARSA, ASB7, ASB8, ASGR1, ASH1L, ASIC1, ASIC3, ASPDH, ASPH, ASTN1, ATAT1, ATF7, ATG12, ATN1, ATOH8, ATP10D, ATP11B, ATP1A4, ATP2B4, ATP5MC2, ATP5MPL, ATP6AP1, ATP6V0A1, ATP7B, ATRN, ATXN1, AXL, AZI2, AZIN2, B3GAT3, B4GALNT3, B4GAT1, B9D2, BACH1///GRIK1-AS2, BAG4, BAHCC1, BAIAP2-DT, BASP1-AS1, BBS5, BBS9, BBX, BCHE, BCL2L11, BCL2L2, BCL6, BCL7A, BDKRB2, BEAN1, BEND5, BGN, BIRC3, BISPR, BLMH, BLOC1S5, BMP1, BMP3, BMP4, BMP5, BMP7, BMPER, BMPR1B, BMT2, BNIP3L, BRD7, BRD8, BRINP2, BRSK1, BRWD1, BRWD3, BTBD3, BTG1, BTG2, BTN2A1, BTN2A2, BTN3A1, BTN3A2, C10orf88, C11orf49, C11orf65, C11orf96, C12orf57, C16orf45, C16orf86, C19orf44, C19orf54, C1orf162, C1orf21, C1orf229, C1orf54, C1QTNF6, C22orf39, C2orf72, C4orf3, C5AR1, C5orf49, C5orf66, C6orf118, C6orf58, C6orf89, C7orf61, C9orf72, CA11, CA14, CA3, CAB39L, CABP7, CACFD1, CACHD1, CACNA1G, CACNA1H, CACNB1, CACNB3, CADM1, CADM3, CADM4, CAHM, CALCOCO1, CALCOCO2, CALU, CAMK1, CAMK1G, CAMK2B, CAMK2N1, CAMKK2, CANT1, CAPN14, CAPN2, CAPN7, CARF, CASC1, CASP7, CAST, CATIP, CBFA2T2, CBFA2T3, CBLB, CBLL1, CBX1, CBX4, CBX5, CBX8, CCDC102A, CCDC125, CCDC130, CCDC136, CCDC148, CCDC159, CCDC167, CCDC183, CCDC184, CCDC189, CCDC33, CCDC62, CCDC74A, CCDC85A, CCDC85C, CCDC92, CCDC97, CCM2, CCNB3, CCND1, CCND2, CCNDBP1, CCNG2, CCNY, CD226, CD24, CD302, CD37, CD8B2, CD99L2, CDADC1, CDC23, CDH10, CDH13, CDH4, CDH6, CDIP1, CDK17, CDK19, CDK20, CDK5RAP3, CDK8, CDKL2, CDKN1A, CDKN2C, CDS2, CDX2, CELF2-AS1, CELF3, CELF5, CELSR2, CEP112, CEP126, CEP170, CEP250, CEP350, CEP97, CERS6, CES3, CES4A, CETN2, CFAP126, CFAP57, CFAP58, CFDP1, CFL2, CGGBP1, CHAD, CHD2, CHP1, CHRD, CHST11, CIART, CIRBP, CITED2, CKAP2, CLASP1, CLBA1, CLCF1, CLCN2, CLCN4, CLCN5, CLDN20, CLEC10A, CLEC16A, CLEC18C, CLEC19A, CLIC5, CLIP3, CLK2, CLPSL1, CLSTN1, CLSTN2, CLSTN3, CLUAP1, CMBL, CMTM1, CMTM3, CMTM5, CMTR2, CNIH1, CNKSR3, CNNM4, CNP, CNPY4, CNTFR, CNTFR-AS1, CNTNAP1, CNTNAP3B, CNTNAP3P2, CNTROB, COL11A1, COL27A1, COL2A1, COL4A1, COL5A1, COL9A2, COLGALT2, COMMD3, COMMD8, COPZ2, COQ10A, COQ5, COQ6, COX15, COX19, CPA1, CPA2, CPA4, CPD, CPEB4, CPED1, CPLANE2, CPM, CPNE3, CPQ, CPSF1, CPT1C, CPXM1, CRB2, CREBRF, CRK, CROCCP3, CRTAC1, CRTC1, CRY2, CRYBA1, CRYZL1, CSNK1G1, CSNK2A1, CSNK2A2, CST2, CST3, CSTF3-DT, CTBP1, CTBS, CTDSP1, CTHRC1, CTSB, CTTNBP2NL, CUBN, CUEDC1, CUEDC2, CUL9, CX3CL1, CXorf40A, CXXC4, CYB561D1, CYB5D2, CYP27A1, CYP2R1, DACH1, DACT3, DARS, DAZAP2, DBH-AS1, DCAF12, DCAF12L2, DCAF5, DCDC2, DCLK2, DCST1, DCST2, DCTN3, DCTN4, DCTN5, DDHD1, DDHD2, DDN, DDOST, DDX47, DENND2A, DENND4A, DENND6B, DET1, DGKH, DHFR2, DHX32, DHX40, DIP2A, DIRC3, DKK1, DKK2, DKKL1, DLC1, DLEC1, DLK1, DLK2, DLX3, DM1-AS, DMGDH, DMXL2, DNAAF3, DNAH6, DNAH7, DNAH9, DNAI1, DNAJC1, DNAJC18, DNAJC27, DNAL4, DNALI1, DNASE1L1, DNHD1, DNM1, DNMBP, DNMT1, DOCK1, DOCK7, DPCD, DPP7, DPYSL4, DPYSL5, DRAM2, DRAXIN, DRC1, DRC3, DTNBP1, DTX3, DUSP10, DUSP18, DUSP3, DVL2, DYM, DYNC1LI2, DYRK1A, DYRK1B, E2F6, EBF2, EBF4, ECE1, ECM1, EDAR, EDN1, EEF1AKMT3, EEF2, EFCAB1, EFCAB12, EFCAB7, EFEMP2, EFHB, EFHC1, EFNB1, EFNB2, EFNB3, EFR3B, EFS, EGLN3, EID2B, EIF3L, EIF4ENIF1, ELF1, ELK4, ELMOD3, ELOVL5, EMID1, EMP3, ENDOV, ENO2, ENO3, ENPEP, ENPP3, ENPP4, ENTPD5, ENTPD6, EPB41L1, EPB41L5, EPC1, EPCAM-DT, EPHA2, EPHA4, EPHB2, EPHB3, EPHX2, ERAP1, ERCC1, ERLIN1, ERLIN2, ESD, ESRRG, EVC, EVL, EXOC4, EXTL1, EYA1, EZH1, F10, F2R, FAIM2, FALEC, FAM107A, FAM13A, FAM149B1, FAM153C, FAM160B2, FAM161B, FAM168A, FAM168B, FAM171A2, FAM172A, FAM178B, FAM183A, FAM193A, FAM198B, FAM201A, FAM206A, FAM20B, FAM210B, FAM214B, FAM219B,

	FAM220A, FAM222A, FAM228A, FAM229B, FAM3D-AS1, FAM47E, FAM53B, FAM53C///LOC100128966, FAM81B, FAM92B, FANK1, FAT4, FAXDC2, FBLN1, FBLN7, FBN2, FBRS, FBXL14, FBXL2, FBXL20, FBXL8, FBXO15, FBXO16, FBXO24, FBXO42, FBXO6, FBXW8, FCER1G, FCHSD2, FCRLB, FEM1B, FER1L4, FEZ1///STT3A-AS1, FGF1, FGF10, FGF17, FGF3, FGFR3, FHIT, FITM2, FKBP1A, FKBP9, FKBP9P1, FLJ12825, FLJ16779, FLJ37453, FLJ46284, FLJ46906, FLNC, FNDC3A, FNDC4, FNDC5, FNTA, FOLR1, FOXA2, FOXB1, FOXD2-AS1, FOXF2, FOXJ1, FOXJ3, FOXK1, FOXO3, FOXS1, FREM1, FRG1BP, FRMD3, FRMD6, FRRS1L, FRS3, FRZB, FSD1L, FST, FSTL3, FTL, FTO, FUNDC2, FUT11, FUT8, FUT8-AS1, FXR1, FXR2, FXYD6, FYN, FYTTD1, FZD1, FZD2, FZD9, GAA, GAB2, GABARAP, GABARAPL2, GABRG3, GAD1, GALC, GALNT16, GALNT5, GALP, GAMT, GAS1, GAS8, GATD1, GBA, GBE1, GCKR, GCNT1, GDAP2, GDF10, GDF11, GDF5, GDI1, GEM, GGT7, GIGYF2, GIPR, GLB1L, GLDN, GLI3, GLI4, GLIPR1, GLIS2, GLUD1P2, GLYATL2, GNAZ, GNB2, GNPDA2, GNS, GOLGA2P10, GPBP1, GPC1, GPC5, GPCPD1, GPHN, GPR132, GPR137C, GPR146, GPR153, GPR155, GPR162, GPR173, GPR182, GPR21///RABGAP1, GPRASP1, GPSM1, GPSM2, GPX7, GRAMD1C, GREB1, GRIN3B, GRINA, GRK5, GSAP, GSE1, GSK3B, GSN, GSN-AS1, GSTA4, GSTK1, GSTM2, GTF2A1, GTF2F1, GTF3C3, GTSE1-DT, GUCA1B, H2AFY2, H6PD, HABP4, HACD2, HAGLR, HAP1, HAPLN2, HCFC2, HDAC6, HEATR5B, HELZ, HEMK1, HERC4, HERPUD2, HES7, HEXIM1, HGC6.3, HGSNAT, HHAT, HHIPL2, HILPDA, HINT3, HIP1R, HIPK1, HIST1H1E, HIVEP3, HLA-DMA, HMGCL, HMGCLL1, HNRNPA1P10, HNRNPUL2, HOMER3-AS1, HOTAIRM1, HOXA-AS2, HOXA-AS3, HOXA1, HOXA2, HOXB-AS1, HOXB-AS3, HOXB1, HOXB7, HOXB8, HOXB9, HOXC4, HOXC8, HPCA, HS1BP3, HS3ST3B1, HSD11B1L, HSD17B11, HSD17B13, HSD17B14, HSD17B8, HSDL1, HSF2, HSPA13, HYDIN, HYMAI, HYPK, IAH1, IAPP, ICAM5, IDH2, IDS, IER5, IER5L, IFFO1, IFI27L2, IFT140, IFT52, IGF2-AS, IGF2R, IGFBP1, IGFBP5, IGFBP7, IGFBPL1, IGIP, IGSF11, IL1RAPL1, IL6ST, ILDR2, IMPG1, ING4, INHA, INMT, INSRR, INSYN2B, INTU, INVS, IP6K2, IP6K3, IPP, IQCD, IQUB, IRX1, IRX2, IRX3, ITFG1, ITGA10, ITGA5, ITGA8, ITGB8, ITIH2, ITPRID2, IWS1, JAM3, JMJD7-PLA2G4B, KAT6A, KATNAL1, KC6, KCNC3, KCNIP2, KCTD1, KCTD13, KCTD16, KCTD18, KCTD19, KCTD21, KCTD7, KCTD9, KDELC1, KDM3A, KDM4B, KIAA0232, KIAA0895, KIAA0895L, KIAA1324, KIAA1324L, KIAA1328, KIAA1755, KIAA1958, KIAA2012, KIF19, KIF26B, KIF27, KIF5C, KIFAP3, KLF10, KLF11, KLF3, KLHDC10, KLHDC8B, KLHL11, KLHL12, KLHL13, KLHL22, KLHL28, KLHL32, KLHL4, KLHL9, KLRF1, KMT5B, KY, KYAT3, LAMB1, LAMTOR3, LAMTOR5, LCA5, LCOR, LDB1, LEF1, LENG8, LEPR, LETMD1, LGALS8, LGALS8-AS1, LGI1, LGR4, LGR5, LIFR-AS1, LIM2, LINC00294, LINC00526, LINC00535, LINC00595///LINC00856, LINC00629, LINC00634, LINC00648, LINC00683, LINC00847, LINC00853, LINC00893, LINC00963, LINC01003, LINC01124, LINC01138///LOC105371225, LINC01144, LINC01152, LINC01285, LINC01456, LINC01473, LINC01549, LINC01571, LINC01686, LINC01703, LINC01749, LINC01801, LINC01869, LINC01933, LINC01948, LINC01977, LINC02035, LINC02043, LINC02175, LINGO3, LIX1, LIX1L, LMAN2L, LMBRD2, LMF1, LMO1, LNPK, LOC100128076, LOC100128164, LOC100130691, LOC100268168, LOC100506142, LOC100506801, LOC100507071, LOC100996724, LOC100996732///LOC107985911, LOC101927521, LOC101927943, LOC101928266, LOC101928414, LOC101929240, LOC101929552, LOC101929704, LOC102723548, LOC102723566, LOC103344931, LOC105369203, LOC105369486, LOC105370526, LOC105371050, LOC105371899, LOC105371925, LOC105375614, LOC155060, LOC254896, LOC284788, LOC339803, LOC646588, LOC730098, LONRF2, LOXL1, LOXL3, LOXL4, LPIN2, LPIN3, LRIG3, LRMDA, LRP2, LRP4, LRRC23, LRRC27, LRRC28, LRRC3-DT, LRRC36, LRRC37B, LRRC43, LRRC4B, LRRC4C, LRRC7, LRRC70, LRRC73, LSAMP, LSM11, LSMEM1, LTA4H, LYPD1, LYRM4-AS1, LYSMD1, LZIC, MAEL, MAGEF1, MALRD1, MAN1A2,

	MAN2A2, MAN2B1, MANBA, MANEA-DT, MAP1A, MAP1B, MAP1LC3B, MAP2K5, MAP2K6, MAP3K1, MAP3K12, MAP7D2, MAPK10, MAPK15, MAPK4, MAPKAPK2, MAPKBP1, MAPRE3, MARCH6, MARCH7, MARCH9, MATN1-AS1, MATR3, MBD5, MBD6, MCF2L, MCFD2, MEAK7, MECP2, MED13, MED13L, MED23, MEG3, MEG8, MEGF6, MEGF9, MEIS2, MEOX1, METRN, METTL6, MEX3A, MGAT3, MIA3, MICAL1, MICALL2, MICU3, MIR4292, MIR503HG, MLLT11, MLLT3, MMP11, MMP2, MN1, MNX1, MOB3B, MON2, MORC3, MORN1, MORN3, MORN4, MORN5, MOSPD2, MPDZ, MPI, MRC2, MRPL30, MRPL46, MRPS6, MRTFB, MSI1, MSI2, MSL1, MSL2, MSLN, MST1P2, MTFR1L, MTHFSD, MTMR3, MTSS1, MTX3, MUC16, MUM1L1, MUT, MVB12B, MXD1, MXD4, MXI1, MYBPC1, MYH8, MYL4, MYL9, MYOF, MYOM3, MYRF, MZF1, NAA35, NAB2, NADK2, NADSYN1, NAPB, NAPSA, NAT8L, NBPF8, NCALD, NCAM1, NCBP2, NCK1, NCOA3, NCOA6, NCS1, NCSTN, NDEL1, NDNF, NDUFB4, NDUFV2, NECAB3, NEK6, NEK9, NEO1, NEPRO, NEU1, NEXMIF, NFAT5, NFATC4, NFIL3, NFKB2, NFKBIL1, NGEF, NGFR, NIPBL, NIPSNAP2, NISCH, NKD1, NKIRAS2, NKX3-2, NKX6-2, NLGN2, NLRC3, NLRC4, NMNAT1, NMT2, NNMT, NOG, NOL3, NOTUM, NPAS2, NPEPPS, NPHP4, NPR3, NPTN-IT1, NPTX2, NR1D2, NR2F1, NR2F1-AS1, NR2F2, NR6A1, NRAP, NRBF2, NRDE2, NRGN, NRN1, NRP2, NRXN3, NSD3, NTAN1, NTF3, NTN1, NUAK2, NUDT17, NUTM2D, NXPH3, OARD1, OBSCN-AS1, OCA2, OGA, OGFOD2, OGT, OLFML2A, OLIG2, OLIG3, OPRL1, OPTN, OR51E2, ORAI3, OS9, OSBPL7, OSBPL9, OSCP1, OTOF, OTUB2, OTUD3, OVGP1, OXSR1, P3H1, P3H3, P3H4, P4HTM, PAAF1, PABPC1, PAG1, PAK2, PAK5, PALLD, PALM, PAPPA, PAQR6, PAQR7, PARP11, PARP16, PARP6, PARVA, PAX3, PBXIP1, PCBP4, PCDH18, PCDH19, PCDH8, PCDHA2, PCDHA3, PCDHB10, PCDHB11, PCDHB13, PCDHB15, PCDHB2, PCDHB3, PCDHB4, PCDHGB7, PCDHGC3, PCED1A, PCNX1, PCP2, PCSK2, PCSK6, PCYOX1, PCYOX1L, PDCD4, PDE1B, PDE6B, PDE8B, PDGFC, PDGFRA, PDK1, PDLIM7, PDRG1, PDYN, PDZRN3, PEG10, PEG13, PELI2, PELI3, PER2, PES1, PEX11A, PEX11B, PEX12, PEX13, PEX19, PFKFB3, PFKFB4, PFN1P2, PGAP1, PGF, PGK1, PGM3, PHC3, PHF2, PHF24, PHF3, PHOX2A, PHPT1, PHYKPL, PI4KAP1, PIAS1, PICALM, PIFO, PIGQ, PIK3IP1, PIK3R1, PJA2, PKDCC, PKNOX2, PLA2G12B, PLA2G6, PLBD2, PLCD1, PLCXD2, PLCXD3, PLD6, PLEKHA4, PLEKHA8, PLEKHB1, PLIN2, PLOD1, PLOD2, PLTP, PLXNA2, PLXNA3, PLXNB3, PMM1, PMP22, PMS2CL, PNCK, PNMA2, PNMA8A, PNN, PNPLA7, PNRC1, POC1B, PODXL, POGK, POGZ, POLK, POLR2B, POLR3GL, POU3F2, POU4F3, POU6F2, PPFIBP1, PPIA, PPIL6, PPM1E, PPME1, PPP1R12B, PPP1R14C, PPP1R32, PPP1R3C, PPP1R3E, PPP1R3F, PPP1R9B, PPP3CB, PQLC1, PRAF2, PRAM1, PRDM12, PRDX2, PRELP, PREPL, PREX1, PRICKLE2, PRKAB1, PRKAB2, PRKAR2A-AS1, PRKCA, PRKCB, PRKCE, PRKRA, PRKRIP1///LOC100630923, PRMT8, PROS1, PROX1, PRPF18, PRR14, PRR3, PRR5L, PRRT2, PRSS35, PRSS53, PRXL2A, PSAPL1, PSD2, PSME1, PSTPIP1, PTAR1, PTCH2, PTGR1, PTGS1, PTP4A2, PTPN13, PTPRM, PTPRN, PTPRVP, PTX3, PURB, PXDC1, PXK, PYGO1, PYGO2, PYROXD2, QKI, R3HCC1L, RAB11FIP4, RAB14, RAB2A, RAB2B, RAB37, RAB40B, RAB4A, RAB5B, RAB6B, RAB7B, RABGEF1, RACK1, RAD23B, RAD52, RAD9B, RALB, RAN, RANBP9, RAP1GDS1, RAP2C-AS1, RAPGEF4, RASA1, RASA4B, RASL10B, RBBP4, RBKS, RBM12B-AS1, RBM20, RBM24, RBM33, RBM43, RBM5, RBMS3///LINC00693, RC3H2, RCN1, RCOR2, RDH5, RDX, RETSAT, RFTN2, RFX2, RFX4, RGMB-AS1, RHBDD2, RHCE, RHOB, RHOJ, RHOU, RIBC1, RILPL1, RIOK3, RIPOR1, RIT1, RLF, RLIM, RMDN1, RMND5A, RND1, RNF11, RNF111, RNF122, RNF130, RNF14, RNF152, RNF157, RNF165, RNF169, RNF19A, RNF214, RNF220, RNF24, RNF38, RNF43, RNGTT, ROR2, RORA, RORA-AS1, RP2, RPA1, RPH3AL, RPL13P5, RPL22, RPL7A, RPLP0, RPRD1B, RPS14, RPS23, RPS4X, RPS6KA3, RPSAP52, RSPH14, RSPH9, RSRC2, RTL1, RTL5, RTN4RL1, RTTN, RUSC2, RXRA, RXRG, S1PR1, S1PR2, SACS-AS1, SAMD10, SAMD8, SARAF, SAT2,

	SAXO1, SCAF4, SCAF8, SCAMP1, SCAMP4, SCAPER, SCCPDH, SCD5, SCHIP1, SCML1, SCML2, SCN2B, SCN4B, SCN5A, SCNN1D, SCP2, SDC3, SDCBP, SDHAF4, SDK2, SEC14L1, SEC14L2, SEC31A, SEC61A2, SECISBP2L, SECTM1, SEL1L, SELENBP1, SELENOP, SEMA3B, SEMA3C, SEMA4F, SEMA4G, SEMA5A, SEMA5B, SEMA6D, SENP5, SENP7, SEPT6, SERINC1, SERPINA1, SERPING1, SERTAD2, SERTAD3, SESN3, SETBP1, SFI1, SFXN1, SGCB, SGCG, SGSM2, SH3BGRL3, SH3BP5, SH3BP5-AS1, SH3D21, SH3GLB1, SH3RF1, SH3RF3, SIAE, SIDT1, SIDT2, SIK2, SIL1, SIM2, SIRPA, SIRT3, SKAP1, SKAP2, SKIDA1, SLAIN2, SLC12A6, SLC16A14, SLC16A4, SLC22A18, SLC22A8, SLC25A14, SLC25A30, SLC25A36, SLC25A37, SLC25A40, SLC26A11, SLC27A1, SLC2A13, SLC2A3, SLC35D2, SLC35E2B, SLC35F1, SLC36A4, SLC37A4, SLC38A3, SLC39A7, SLC41A3, SLC43A2, SLC4A8, SLC51B, SLC5A3, SLC6A1, SLC6A16, SLC6A8, SLC7A11, SLC7A11-AS1, SLC8A2, SLC8A3, SLC9A3-AS1, SLC9B1, SLFN5, SLIT1, SLIT2, SLIT3, SMAD2, SMAD5, SMAGP, SMAP2, SMARCA1, SMARCC2, SMARCD3, SMG1P7, SMG6, SMG9, SMIM17, SMIM29, SMIM32, SMOC1, SMOX, SMPD1, SMPD3, SMYD2, SMYD3, SNAI2, SNAPC1, SNORD6, SNRNP48, SNW1, SNX18, SNX2, SNX29, SNX3, SNX30, SNX32, SOAT1, SOBP, SOCS5, SOGA1, SORCS2, SOX1, SOX1-OT, SOX11, SOX21, SOX5, SOX8, SP1, SP5, SPA17, SPACA9, SPAG17, SPAG6, SPAG8, SPATA7, SPATS2, SPEN, SPICE1, SPIN3, SPSB1, SPSB4, SPTY2D1, SRCIN1, SRP54, SRP72, SRPK2, SRR, SRSF12, SSC5D, SSPO, SSTR3, ST3GAL3, ST3GAL5, ST6GALNAC5, ST6GALNAC6, ST8SIA2, ST8SIA4, STARD4, STARD4-AS1, STARD9, STAT1, STAT5B, STK24, STK3, STOML3, STON1, STPG1, STRA6, STRADB, STT3B, STX12, STX17, STX1A, STX7, SUCLG2, SUCNR1, SUFU, SUGCT, SUGP2, SUMF2, SUPT3H, SUPT6H, SUPT7L, SUSD6, SVOP, SYCE3, SYNGR1, SYNJ1, SYNPO, SYNPR-AS1, SYS1, SYT11, SYT17, TAB2, TADA2B, TAGLN3, TAOK1, TAOK2, TAPBPL, TAPT1, TAS2R5, TBC1D10C, TBC1D19, TBC1D32, TBC1D9B, TBX15, TCAF1, TCEA2, TCF12, TCF4, TCP11, TCP11L1, TCTE1, TDRD3, TEAD1, TECPR1, TEF, TEKT2, TEKT5, TENM3, TENM4, TENT4A, TEP1, TERF2IP///LOC105371348, TET1, TET2, TEX22, TF, TFAP2B, TGFB2, THAP8, THBD, THBS3, THBS4, THG1L, THPO, TIGD1, TIMP1, TIMP3, TINCR, TIPARP, TLE6, TM2D1, TM6SF2, TM9SF2, TM9SF4, TMC7, TMCC3, TMED4, TMED8, TMEM101, TMEM106A, TMEM106B, TMEM107, TMEM120A, TMEM123, TMEM128, TMEM130, TMEM132A, TMEM132E, TMEM136, TMEM167B, TMEM168, TMEM169, TMEM170B, TMEM175, TMEM179B, TMEM182, TMEM204, TMEM219, TMEM221, TMEM230, TMEM248, TMEM25, TMEM255A, TMEM263, TMEM35A, TMEM38B, TMEM42, TMEM44, TMEM45A, TMEM87B, TMEM88, TMEM8B, TMEM91, TMEM94, TMEM98, TMF1, TMLHE, TMPRSS5, TMTC2, TMUB2, TMX4, TNFAIP1, TNFRSF10B, TNFRSF19, TNIP1, TNNC1, TNP1, TNS3, TNXB, TOGARAM1, TOM1L2, TOX4, TP53BP1, TP53BP2, TP53I11, TP53INP1, TPGS2, TPI1, TPM2, TPPP3, TPT1-AS1, TRADD, TRAF5, TRAF6, TRAK1, TRAPPC11, TRAPPC5, TRIL, TRIM16, TRIM17, TRIM2, TRIM41, TRIM44, TRIM55, TRIM63, TRIM68, TRIM8, TRIP4, TRIQK, TRPM8, TRPV3, TRPV4, TSC1, TSC22D1, TSGA10IP, TSHZ1, TSHZ2, TSPAN14, TSPAN18, TSPAN3, TSPAN31, TSPAN32, TSPAN5, TSPAN6, TSPAN7, TSPOAP1, TSPYL2, TTC25, TTC37, TTC4, TTC9C, TTLL1, TTYH2, TUB, TUBG2, TULP3, TULP4, TUSC3, TUT4, TVP23A, TXNIP, TYK2, TYRO3, U2AF1///U2AF1L5, UBAP2L, UBE2L6, UBE2R2, UBL3, UBQLN2, UBQLN4, UCN, UFC1, UFL1, UHMK1, UHRF2, ULK2, UNC119B, UPK2, USP11, USP20, USP3, USP3-AS1, USP30, USPL1, UVRAG, UXT, VAMP3, VAMP5, VASH1, VAX2, VEZF1, VIM, VIPAS39, VPS13C, VPS16, VPS28, VPS33B-DT, VPS9D1, VSTM2L, VTN, VWA5A, WASF3, WBP1, WDFY3-AS2, WDR13, WDR26, WDR27, WDR31, WDR54, WDR60, WDR66, WDR88, WDTC1, WDYHV1, WHRN, WIPF1, WIPF2, WLS, WNT3///LOC101929777, WNT5A, WNT8A, WRB, WSB1, WWTR1, XKR5, XKR7, XPNPEP1, XRCC1, XRN1, YAF2, YAP1, YIPF5, YIPF6, YPEL1, YPEL2, YPEL5, YWHAE, YWHAH, YY1, ZBTB10,

	ZBTB21, ZBTB25, ZBTB26, ZBTB41, ZBTB5, ZBTB7C, ZC3H11A///ZBED6, ZC3H6, ZC3HAV1L, ZCCHC14, ZCCHC24, ZDHHC18, ZDHHC4, ZDHHC9, ZEB2-AS1, ZFAND3, ZFAS1, ZFC3H1, ZFP1, ZFP14, ZFP2, ZFP36L2, ZFP90, ZFP91, ZFYVE1, ZIC2, ZIC3, ZKSCAN1, ZKSCAN2, ZKSCAN4, ZKSCAN8, ZMYM3, ZMYND10, ZMYND12, ZNF106, ZNF12, ZNF136, ZNF148, ZNF182, ZNF2, ZNF227, ZNF236, ZNF25, ZNF250, ZNF251, ZNF264, ZNF276, ZNF316, ZNF322, ZNF324B, ZNF329, ZNF333, ZNF343, ZNF346, ZNF354C, ZNF385A, ZNF385C///C17orf113, ZNF404, ZNF423, ZNF436, ZNF438, ZNF441, ZNF460-AS1, ZNF467, ZNF474, ZNF484, ZNF497, ZNF503, ZNF503-AS2, ZNF516, ZNF521, ZNF529, ZNF548, ZNF554, ZNF555, ZNF561, ZNF571, ZNF585A, ZNF599, ZNF624, ZNF627, ZNF629, ZNF654, ZNF664, ZNF678, ZNF692, ZNF695, ZNF699, ZNF70, ZNF703, ZNF704, ZNF709, ZNF710, ZNF710-AS1, ZNF764, ZNF805, ZNF816, ZNF823, ZNF843, ZNF85, ZNF862, ZNRF3, ZRANB2-AS2, ZSCAN1, ZYG11B
3	*ABCE1, ABCG2, ABCG4, ABI3BP, ACADL, ACAN, ACAP3, ACHE, ACKR3, ACP4, ACSBG1, ADAM19, ADAM9, ADAMTS1, ADAMTS12, ADAMTS17, ADAMTS18, ADAMTSL5, ADAP2, ADCY9, ADCYAP1R1, ADGRA2, ADGRD1, ADGRG6, ADGRL4, ADRA1D, ADRA2A, AFAP1L1, AGPAT5, AGRN, AHCTF1, AHNAK2, AK3, AKR1C1, ALDH1A3, ALDH1L2, ALDH8A1, ALDOA, ALK, ALOX12, ALPK2, ALPL, ANGPT2, ANGPTL2, ANKRD1, ANKRD33B, ANKRD50, ANXA1, ANXA4, ANXA5, AP5B1, APLN, APOA1, APOL6, APOO, AREG, ARHGAP11A, ARHGAP17, ARHGAP26, ARHGAP28, ARHGAP30, ARHGAP36, ARHGAP42, ARHGDIA, ARHGDIB, ARHGEF12, ARHGEF3, ARHGEF6, ARPC2, ASAH1, ASAP1, ASCC3, ATAD3C, ATF3, ATP12A, ATP2B2, ATP5F1C, ATP6V0D2, ATP6V1C1, ATRNL1, B4GALNT1, B4GALT1, B4GALT5, BAALC, BAALC-AS1, BACE2, BAG2, BAG3, BCAR3///MIG7, BCL3, BCYRN1, BHMG1, BLOC1S3, BMP1, BMP8B, BRK1, BTG3, C16orf72, C1GALT1, C1QL2, C1QTNF1, C1R, C20orf144, C22orf23, C2CD4C, C2orf72, C5orf56, C8orf34, CA8, CACNG5, CALHM2, CALHM5, CAMTA2, CAP1, CAPN9, CARD14, CARD6, CAV1, CAV2, CAVIN1, CBL, CCDC102B, CCDC93, CCNO, CCSER2, CD109, CD300LG, CD59, CD81, CD82, CDC14B, CDC42BPG, CDC42EP1, CDC42EP3, CDC42EP4, CDH11, CDIPTOSP, CDK2, CDK6, CDKL5, CDKN1A, CDKN2A, CDKN2B, CDKN2D, CDR2, CELP, CEMIP, CEP104, CEP85L, CHI3L2, CHPT1, CHRNA7, CHST1, CHST15, CHST3, CISH, CIT, CLDND1, CLEC2L, CLIC1, CLIC2, CLIP2, CLU, CNN3, CNTNAP2, COL15A1, COL5A2, COL5A3, COL8A1, CORIN, COTL1, COX1, COX4I2, COX7A1, CPEB2, CPNE5, CPOX, CPXM2, CREB3L1, CREB5, CRHBP, CRIM1, CRISPLD2, CRYBB1, CRYBG3, CS, CSMD3, CSPG4, CSRNP1, CTF1, CTNNB1, CTTN, CTTNBP2, CXCL1, CXCL14, CXCL16, CXCL2, CXCL5, CYB5R1, CYGB, CYP11A1, CYTH3, DAAM2, DAZAP2, DAZL, DCAF10, DCBLD1, DCTN2, DENND2D, DENND3, DEPP1, DERL1, DGKA, DHH, DHRS7, DIO3, DIP2C, DIRAS2, DLL4, DNAH10, DNAJB1, DNAJC3, DNAJC6, DNAJC8, DNASE1L2, DOK6, DRAM1, DRD2, DSC3, DSCAM, DTX4, DUOX2, DUSP14, DUSP16, DUSP8, DYNLL2, DYNLT1, DYRK2, EBF1, EBI3, EDEM1, EDNRB, EEPD1, EFNA1, EHBP1L1, EI24, EIF4A2, ELK3, ELL2, EMC7, EMILIN2, EMP1, EMP3, EN2, ENDOD1, ENTPD2, EP400, EPAS1, EPB41L4A-DT, EPHA3, EPSTI1, ERAP2, ERFE, ERO1A, ESRRB, ETS1, ETV7, EVA1A, EVA1C, EXOC8, EXPH5, F2RL1, F3, F5, FABP7, FAM110D, FAM114A1, FAM160A1, FAM184B, FAM210A, FAM219A, FAM234B, FAM43A, FAM49A, FAM50A, FAM57A, FAM91A1, FAR2P2, FARP2, FAXC, FBH1, FBLIM1, FBXL7, FBXO3, FBXO45, FEM1C, FGD5, FHL3, FJX1, FKBP14, FLI1, FLOT1, FLT1, FLT4, FMN1, FN1, FNBP1, FNDC3B, FOSL1, FOSL2, FOXB2, FOXC1, FOXC2, FOXI1, FOXI3, FOXJ2, FOXL1, FOXN4, FOXQ1, FRMD4A, FRMD4B, FUBP3, FZD6, GABBR2, GABRP, GADD45A, GADD45G, GANAB, GAS6, GAS7, GATA3, GBGT1, GBX2, GDNF, GDNF-AS1, GFM1, GFPT1, GFRA2, GIPR, GJC1, GLG1, GLP1R, GNA11, GNAL, GNAO1, GNB4, GNLY, GPR137B, GPR139, GPR150, GPR157, GPR4, GPR68, GRID1, GRIP2, GRM8,*

	GSG1L, GUCY1A1, GUCY1A2, GUCY1B1, GXYLT1, HACD3, HACD4, HBEGF, HCN3, HEBP1, HECA, HERC2, HERPUD1, HES2, HES4, HES5, HEXB, HEY1, HEYL, HIC1, HIF1A, HIGD1B, HIPK3, HIST1H2BK, HIVEP2, HKDC1, HMGB2, HOPX, HPSE2, HRH1, HS6ST1, HSD17B2, HSPA4L, HSPB8, HUNK, IBTK, ICE1, ID4, IDH3A, IER3, IFFO2, IGF1R, IGF2BP1, IGSF3, IL11, IL12A, IL13RA1, IL15RA, IL1R1, IL1RAP, IL4I1, IL7, IMPAD1, INHBB, INPP1, INPP5D, INSYN2, IPO7, IPO8, IREB2, ITCH, ITGA1, ITGA3, ITGA9, ITGB1, ITGB3, ITPKC, JAG1, JAK1, JAK2, JCAD, JMJD6, JPH2, KAZN, KCNA1, KCNA5, KCNA6///GALNT8, KCNAB1, KCNC1, KCNF1, KCNH1, KCNH8, KCNIP3, KCNJ1, KCNJ5, KCNJ8, KCNK17, KCNN3, KCNQ4, KCNT1, KCTD12, KERA, KIAA1147, KIAA1191, KIAA1549L, KIF13A, KIF13B, KIFC3, KIRREL1, KITLG, KLC2, KLF6, KLF7, KLHL29, KLHL3, KLHL6, KPNA1, KREMEN1, KRT16, KTN1, L1CAM, LAMA4, LAMC1, LAMP2, LAMP3, LAMP5, LARP6, LAYN, LBH, LBX2, LEO1, LGALS3, LGALS9, LGALSL, LGI2, LHCGR, LHFPL6, LIF, LIFR, LIMCH1, LIMK2, LINC00310, LINC00475, LINC00698, LINC00857, LINC00941, LINC01198, LINC01291, LIPA, LIPG, LMCD1, LOC100505635, LOC100506885, LOC100507560, LOC101928100, LOC101929524, LOC101929798, LOC102724474, LOC102724566, LOC102724957, LOC105369201, LOC105376323, LOC105376567, LOC107986197, LOC152048, LOC283922, LOC400464, LOC729732, LONRF1, LOX, LPAL2, LPGAT1, LRCH1, LRP10, LRP1B, LRRC10B, LRRC25, LRRC32, LRRC59, LSP1, LSS, LTBP2, LTBR, LTN1, LURAP1, LURAP1L, LYN, LYSMD3, MAF, MAFB, MAFG, MAFK, MAGIX, MAL, MAML2, MAP3K8, MAPK1, MAPK11, MAPK8IP2, MARCKS, MASP1, MCAM, MCL1, MCOLN3, ME1, MECOM, MEGF8, MEST, METRNL, MGAT4B, MICAL3, MID2, MIR4435-2HG, MIR573, MIR9-3HG, MISP, MKI67, MLC1, MLF2, MLX, MMD, MMP19, MMP7, MMP9, MOCS1, MON1B, MOV10L1, MPPED1, MRGPRF, MSLN, MSMO1, MST1L, MTCP1, MTDH, MTMR10, MTRF1L, MXRA8, MYL12A, MYL12B, MYL3, MYO10, MYO16, MYO1F, MYO5B, MYOM2, MYORG, MYPOP, MYT1, NACAD, NACC2, NALCN, NAPG, NAT1, NAT8L, NCKAP1, NCOR2, NDFIP1, NDUFS1, NECAP1, NECTIN4, NEURL1B, NFATC1, NFIC, NFKB1, NFKBIA, NFX1, NFXL1, NID1, NIPAL2, NMD3, NMUR1, NOLC1, NOMO3, NORAD, NOV, NOX1, NPC1, NR3C1, NRARP, NRP1, NRSN2, NSMAF, NT5E, NTN3, NUDT4///NUDT4B, NUP205, NXN, OAF, OLFM4, OSBPL11, OSBPL3, OSBPL6, OSGIN1, OSMR, OSR1, OSTF1, OSTM1, OTUD4, OTUD7B, P2RY11, P2RY2, PABPC4, PAEP, PAF1, PALM2-AKAP2, PAQR3, PARD6G, PARM1, PARP14, PCDH10, PCDHA8, PCDHGA11, PCDHGA12, PCNP, PCSK5, PCYT1A, PDE1C, PDE3A, PDE4C, PDE4C, PDE6C, PDGFB, PDHA1, PDIA2, PDK4, PDP1, PDZD2, PELI1, PFDN1, PFKFB2, PGM5, PHF11, PHF19, PHLDA3, PHLPP2, PHRF1, PI4KA, PIGH, PIGZ, PIK3CA, PITRM1, PLA2G2A, PLAC8, PLAU, PLB1, PLBD2, PLCD3, PLEKHA2, PLEKHH3, PLEKHM1, PLEKHN1, PLK2, PLLP, PLP2, PLVAP, PLXDC1, PLXNA2, PNMA6A, POLH, POLR1C, POM121C, POMC, PON3, POPDC2, POPDC3, PPFIA1, PPIC, PPP1CB, PPP1R12C, PPP1R16B, PPP1R3D, PPP2R5B, PPP3CC, PRAG1, PRCD, PRDM16, PRKAR2A, PRR16, PRRG1, PRXL2C, PSMB10, PSME4, PSMF1, PTGDR2, PTGFRN, PTGIS, PTH1R, PTHLH, PTPN12, PTPN18, PTPN3, PTPRE, PTPRK, PTPRU, PVR, PXDN, PXDNL, PXYLP1, PYGL, PZP, RAB11FIP1, RAB32, RAB5C, RAB6A, RAB8B, RABEP1, RABGGTB, RAI14, RALGAPB, RASA3, RASGEF1C, RASL10A, RASSF4, RASSF5, RCAN1, RCL1, RELB, REM2, RFK, RFTN1, RGCC, RGS11, RGS19, RGS20, RGS4, RGS6, RHBDF2, RHCG, RHOBTB3, RHOC, RHOG, RHOQ, RHOV, RHPN2, RIN3, RIPK4, RIPOR3, RLN1, RMDN3, RNF128, RNF144A, RNF168, RNF180, RNF19A, RNF19B, RNF6, RNH1, RNPEPL1, ROPN1B, ROPN1L, RPS3A, RPS6KA2, RRAGD, RUFY3, RUNX1///LOC100506403, RUNX3, S100A3, SAMD12, SAR1B, SATL1///LOC105369163, SAYSD1, SCG2, SCGB1A1, SCGB3A2, SCTR, SDC1, SDC2, SEC23A, SEC24A, SEC61A1, SELPLG, SENP2, SEPT11, SEPT8, SERHL2, SERINC3, SERP1, SERPINB6, SERPINB9, SERPINE1, SESN2, SFRP2, SFTA1P, SGPP1, SGTB,

	SH3BP4, SH3KBP1, SH3PXD2B, SH3TC2, SHISA3, SLC11A1, SLC12A2, SLC13A5, SLC16A7, SLC1A4, SLC22A23, SLC23A2, SLC25A18, SLC25A20, SLC25A32, SLC25A51, SLC2A1, SLC2A6, SLC34A2, SLC35E1, SLC35F2, SLC35F6, SLC35G2, SLC36A1, SLC45A4, SLC46A3, SLC4A2, SLC50A1, SLC6A17, SLC7A1, SLC7A10, SLC7A5, SLC7A7, SLC8A1, SLC9A2, SLC9A9, SLCO2B1, SLFN12, SLITRK2, SMARCA5, SMOX, SMTNL2, SMURF2, SNORA73B, SNRK, SNTG2, SNX10, SNX13, SNX19, SNX27, SOCS7, SOST, SOWAHB, SOX18, SOX7, SOX9, SP140L, SPATA8, SPHK1, SPINK1, SPON1, SPON2///LOC100130872, SPPL3, SPTLC3, SQSTM1, SRL, SRPRA, SRPX, SSH1, SSR2, SSR3, ST3GAL1, ST5, STAM2, STARD5, STIP1, STK10, STK17B, STK26, STK32B, STK40, STMN2, STX3, SULT4A1, SYN1, SYNM, TACC1, TAF1D, TAGLN2, TBX18, TBX3, TCIRG1, TCN1, TDO2, TEK, TENT5C, TESK1, TEX14, TEX29, TFF1, TFPI, TFRC, TGFB1, TGFB1I1, TGM1, TGM2, THBD, THRB, TIMM21, TIMP2, TINF2, TKTL1, TM4SF1, TM4SF19, TM4SF19-AS1, TM9SF3, TMBIM1, TMC4, TMCC2, TMED5, TMED7, TMEM100, TMEM109, TMEM117, TMEM127, TMEM131L, TMEM135, TMEM144, TMEM154, TMEM171, TMEM184A, TMEM200B, TMEM229A, TMEM255B, TMEM40, TMEM43, TMEM51, TMEM51-AS1, TMEM63C, TMEM64, TMEM71, TMEM9B, TMOD1, TMOD3///LOC112268148, TMSB4X, TMX3, TNFAIP2, TNFRSF10C, TNFRSF10D, TNFRSF21, TNFSF10, TNFSF4, TNNT3, TNS2, TOMM70, TOR1B, TOX2, TP53INP2, TPBGL, TPCN1, TPCN2, TPPP, TPRG1, TRAPPC6B, TRH, TRIB1, TRIM22, TRIM26, TRIM45, TRIM47, TRIM69///LOC100419583, TRIM7, TRPC6, TRPS1, TSPAN1, TTF2, TTYH1, TVP23B, TXNDC11, UACA, UAP1L1, UBA2, UBASH3B, UBE2D2, UBE2I, UBE2Q2P1, UBE3C, UGGT1, UNC5B, UPB1, UPP1, URB1, USP7, VAMP8, VAT1L, VAV3, VCAM1, VCPIP1, VDAC1, VEPH1, VGLL2, VPS13D, VPS18, VPS37A, VSIR, VSTM4, WFDC2, WFIKKN2, WNK4, WNT6, WNT7A, WNT7B, WNT9A, WWP1, XKR4, XPO5, XRN2, YBX3, ZBTB42, ZCCHC12, ZDHHC1, ZFAND5, ZFP36, ZHX2, ZNF207, ZNF750, ZYX

Table S2. Lists of overexpressed and down-regulated genes from comparison groups.

Comparison Group	Condition	List of Genes
CONTR-NICD	Up-regulated	*LAMP5, MMP9, CCN3, SLC1A4, FLT1, RUNX3, RGS4, PLA2G2A, PLXDC1, CORIN, OLFML2B, ACSBG1, RHOV, RHCG, LBH, ANGPT2, MCAM, CHST1, KCNA5, MAFB, SFTA1P, GABRP, GDNF, POMC, MASP1, ALPK2, HBEGF, DEPP1, SLC6A12, HCK, MYORG, TCN1, VAMP8, PPEF1, CYTOR, CCDC192, CHI3L2, DAAM2, VEPH1, ADAMTS18, AGT, HSPB8, TMEM100, ENDOD1, MIR4435-2HG, HEY1, EVA1C, IL1R1, SERPINB9, EMP1, CPXM2, SLC45A4, MME, OLFM4, ZNF750, LIFR, CACNA2D1, PPIC, OSMR, RGS6, IRF4, CXCL5, EMILIN2, ADAMTS15, TRPC6, ANKRD1, NRP1, FOXC1, PNPLA5, KCNN3, ENTPD2, FOXQ1, TNFAIP3, ATP6V0D2, EFHD1, IFFO2, TAL1, SMTNL2, ID4, CRISPLD2, ADGRA2, TKTL1, LPL, IRF8, JAK1, LAYN, SGK1, NGF, LGALS3, CASP17P, GPR139, DTX1, EBF1, PDE3A, EPHX1, SCGB3A2, MCHR1, CREB5, SRPX, SNAI2, KCNK17, FAM49A, MMP13, RGCC, PDZD2, TFF1, PAPSS2, OAF, KERA, PTH1R, ATP12A, ADAMTSL4, CAV2, ANKRD33B, TRIML2, HES5, PLD1, ARHGAP42, GFRA2, IL12A, EFNA1, SFXN3, NECTIN4, SORBS2, TGFB1I1, GLP1R, SLC7A5, EPHA3, FURIN, HEYL, FN1, COL5A3, UAP1L1, KIAA1549L, INHBB, WFDC2, MPPED1, LAMC2, PGM5, ST14, MMP7, CDC42EP1, NRARP, SH3KBP1, MGAT4C, FHDC1, PCDH10, LRRC10B, CD82, ITGB3, TUBA4A, IGFBP3, FOXI1,*

		MXRA8, TNNT3, IFI16, AFAP1L2, SPON1, CAV1, IRF6, PMEPA1, LRRC32, COL8A1, GUCY1B1, KCNA1, KRT16, ARHGEF28, MYOM2, CACNG5, SCGB1A1, AFAP1L1, EPAS1, VAV3, EXPH5, CDH11, STMN2, PROK1, USP43, FBLN5, KCNE4, GABBR2, ETS1, SSUH2, KRT6B, SVIL, SOX9, LOX, CYGB, F2RL1, FABP7, ALDH1A3, MMP14, ITGA1, MPP1, SOX18, GAS7, B3GNT7, ACKR3, PELI1, EOGT, SRL, CISH, GPC1, TGFB1, RFTN1, CABP1, OSTF1, PDZK1IP1, IGFL1, GLIS3, TNFSF10, COBLL1, CALHM5, ADAP2, SLC13A5, SHISA3, LAMA4, KRT6A, ADGRF2, HTR1F, NOS1, PLP2, SAT1, ITM2A, FRMD4A, ADAMTS12, IGF2, COL5A1, EEPD1, LAMC3, ITIH5, ERFE, SCG2, MT1X, JAK2, RASSF4, MCTP1, CSPG4, TNFRSF21, BHMG1, CAPN9, RASGRP1, SLC7A7, NTSR2, NOTCH1, THSD4, PLA2G5, CXCL12, NIPAL1, KRT14, LOC101929798, SLFN13, LMNA, SPINK1, DUSP15, ALDH1A2, TAGLN2, NIBAN1, TMEM63C, PPM1H, PLEKHG1, BBS5, LPGAT1, GATA3, HSD17B2, KRT5, JPH2, TM4SF19, IFIT3, ADAM19, DENND2B, SPATA8, MYC, STX3, SAMD12, CCR1, DPF3, WNT7B, CPEB2, MBP, IFIT2, TNS2, SPARCL1, HR, PAEP, NALCN, GRIP2, TEK, COL5A2, DMD, ARHGEF3, STK32B, CDKN2B, CABLES1, ELF4, LGI2, ERO1A, FAM234B, GJA4, IER3, CXCL14, FOSL2, MBOAT1, GRIN2D, TMBIM1, HRH1, KALRN, PMAIP1, ALDH1L2, PALMD, FZD1, KCNF1, DKK3, IL1RAP, JCAD, S100A3, LSP1, SOCS2, HAPLN3, CRHBP, APOA1, MECOM, KCNQ4, TRIM22, UNC5B, CDC14B, LINC00461, PLCXD3, LINC00346, CLIP2, CHST15, SYK, TMEM51, GABRB3, PLEKHA2, ITGA9, CRYBB1, AFP, ALOX12, HSD11B2, BCL11A, ANKRD50, TPPP, NR3C1, MMP25, RARRES2, HES2, SERPINB6, PTGIS, TMEM144, NEURL1B, SLC22A23, LYST, FGD5, ELL2, ADGRB3, LINC01198, MYL3, MEGF10, KIAA0040, SPON2, ROBO2, SLCO2B1, TENT5C, ITGA3, RHBDL3, ESRG, LINC00698, MMP12, F12, IL15RA, FOXS1, PLXNA2, NMNAT2, MAPK13, PCSK5, C2CD4C, SLIT3, SPATS2L, IFNGR1, LINC01278, ANXA2, PDE1C, DSC3, FYB1, CLIC2, SLC8A1, PRNP, EBF4, THRB, THBD, NUDT4, CXCL1, MYOZ3, TGM2, PEAR1, ADGRF4, SYP, IL10RA, TBX18, BNC1, SLC25A18, SNRK, TRIB3, CEMIP, XPO5, CAPG, TMEM163, JAG1, LYSMD3, VCAM1, PRDM16, MPPED2, PLXND1, MARC2, TMEM64, SORBS1, ADAM9, UPP1, ZFP36, TTYH1, TMEM268, RNF128, CSMD3, LCK, DENND2D, PHLDA2, EDNRB, CCDC102B, KRT17, IL32, PLAT, ARHGEF6, DIRAS2, KLF9, NKAPL, CD44, ADGRD1, LOC101927359, PPARGC1B, ADORA2B, CDH1, MAFK, SLC12A2, KDR, SH2B2, TRIM47, ESYT3, FJX1, P2RY2, FOSL1, DOCK11, TCIRG1, NID1, CDC42EP3, GBP1, ACSL6, SLC7A10, SLC46A3, MYO3A, ASAH1, VSNL1, RNF168, ARHGAP28, PRR16, COL18A1, MYO5B, COX4I2, PHLDA1, LIPG, TES, EMX2OS, KCNAB1, TRIB1, PLAAT5, SHANK2, PLK2, IRF1, CHPF, LPAR3, MAF, TRAF3IP2, KLHL3, FAM241A, ESRP2, WNT9A, CTTN, GBX2, ANK1, CD109, SYTL3, RPS6KA2, DAAM2-AS1, OSGIN1, TEC, CD59, NFATC1, FAM114A1, DUSP5, CGNL1, RCL1, CASS4, PTGFRN, KLHL6, KRT75, EN2, LMCD1, LINC02154, SESN2, OSBPL3, FRMD4B, DHRS3, POU2AF1, LINC01168, EEF1A2, GUCY1A2, KLHL29, IDH3A, ADARB2, NXN, C15orf39, STAT5A, DIRAS1, GRID1, GSG1L, MOCOS, TP53AIP1, RHOC, PDGFRB, ESR1
	Down-regulated	*TNNC1, EGR2, MAP2, C6orf141, CALB1, PRMT8, KLHDC8A, S100B, NR2F2, CAMK2A, TNP1, KCNK12, NRGN, TFAP2B, PRRT2, EFCC1, TFDP2, SCD5, C6orf118, FGF10, LOC105375304, PLEKHA7, MAP6,*

		FAM92B, PIMREG, TSPAN7, LINC00476, FLRT3, TMEM106C, PIK3R3, EID2B
SH-NICD	Up-regulated	*LAMP5, PLXDC1, MMP9, CORIN, PLA2G2A, ACSBG1, CCN3, RHOV, GABRP, TAL1, KCNA5, LBH, TCN1, OLFML2B, RGS4, OLFM4, RUNX3, POMC, SFTA1P, MYORG, CCDC192, PPEF1, CYTOR, DAAM2, MAFB, HEY1, DEPP1, EVA1C, DUSP15, ANGPT2, VEPH1, KCNN3, ADAMTS18, HCK, TMEM100, MMP13, LIFR, KCNE4, MCAM, CPXM2, SLC1A4, SLC45A4, RGS6, CHST1, IL1R1, DTX1, RFTN1, FOXC1, PLD1, SLC6A12, KRT17, EFNA1, LAYN, HES5, ADGRA2, IRF4, TKTL1, CASP17P, ACKR3, GADD45G, PMEPA1, CHI3L2, EFHD1, EBF1, TFF1, MYOM2, ENTPD2, IL12A, PNPLA5, PDZD2, FLT1, GDNF, PTH1R, MIR4435-2HG, GPR139, TRPC6, KCNK17, TNFAIP3, KERA, FOXQ1, ENDOD1, INHBB, RHCG, ZNF750, EMILIN2, PLCXD3, SMTNL2, EPHX1, NRP1, HBEGF, SCGB1A1, IRF8, SCGB3A2, FOXI1, NOS1, BBS5, SPON1, EBF4, MMP7, MGAT4C, MXRA8, MME, MCHR1, PCDH10, EPHA3, ITGB3, ID4, CACNG5, FABP7, FN1, HTR1F, HEYL, RASGRP1, PROK1, LRRC10B, MPPED1, ADAMTS15, CRISPLD2, ABCA4, ADAP2, FZD1, JPH2, OSMR, KRT16, VAMP8, SERPINB9, SHC2, PPIC, KRT14, GAS7, ARHGEF28, NGF, IGF2, HSPB8, LINC00461, NRARP, GLP1R, IGFL1, TNFSF10, IFFO2, HR, CISH, LAMA4, CLDN5, PGM5, CDH11, MASP1, KRT6B, SCG2, HSD17B2, DMD, PDE3A, LAMC2, NTSR2, SEMA5A, LINC01198, PLA2G5, GUCY1B1, GFRA2, SRL, HAPLN3, MT1X, COL5A1, RASSF4, JAK2, KRT6A, B3GNT7, SH3KBP1, PDZK1IP1, AFAP1L2, FBLN5, LRRC32, THBD, TRIM55, LAMC3, HRH1, GLIS3, TM4SF19, IFI16, SNAI2, CCR1, TNNT3, ALDH1A2, SOX18, SHISA3, BHMG1, C2CD4C, IRF6, SPARCL1, COL8A1, CGNL1, FAM107A, EXPH5, CRYBB1, ADAMTS12, PALMD, CACNA2D1, THSD4, MBP, FAM49A, FHDC1, SMOC2, NALCN, VSTM4, PELI1, JAK1, SPATA8, KRT5, ITGA9, STMN2, SVIL, NOTCH1, CEMIP, DPF3, GPC1, TTYH1, ANKRD33B, SNTG2, IFIT2, P2RY2, WNT7B, TMEM63C, LPL, KRT75, ST14, POPDC2, SLC22A5, MCTP1, SLC22A23, SH2B2, GJA4, TGFB1I1, HES4, USP43, GABBR2, UAP1L1, TENM4, PLEKHG1, ADAMTSL4, MMP14, RGCC, MYL3, IFIT3, RNF152, GATA3, KIAA1549L, LINC00346, CXCL5, SLC7A7, LMCD1, LOC101929798, SLIT3, KLHL29, SYTL3, MMP12, CXCL12, CYGB, KLHL3, TC2N, NECTIN4, ADRA2A, NMNAT2, ADGRB3, PLXNA2, FAM234B, ALPK2, COBLL1, CAPN6, CAPN9, ADGRF2, SOX9, CD82, SEMA5B, AGT, STK32B, PAPSS2, PAEP, CXCL2, XPO5, POU2AF1, ITGA1, IL10RA, PLEKHF1, BMPR1B, C8orf34, KALRN, TMEM163, SLC13A5, ADGRD1, TMBIM1, SAT1, OSGIN1, STX3, CABP1, FURIN, MATK, TOX2, IFNGR1, MEGF10, IRF1, JCAD, NKAPL, EEPD1, DIRAS1, RPS6KA2, TMEM144, CXCL1, CALHM5, MAF, PPM1H, FAM110D, SLC46A3, DKK3, CLIP2, TNFRSF9, JAG1, KCNA1, WFDC2, KCNQ4, LPGAT1, ACSL6, FOXF1, SAMD12, C11orf88, GBP1, ADGRF4, GRIN2D, DAAM2-AS1, OSTF1, SLC25A18, DENND2B, TMOD1, WNT9A, CTTNBP2, EMP1, EMX2OS, FBXL7, ARHGAP42, KCNAB1, CDH1, GPR55, ARHGAP28, TMEM158, NFATC1, CDC42EP1, HPSE2, SLC38A8, PTGIS, LOC101927359, SERHL2, IL32, CAV2, KCTD12, SLCO2B1, SPINK1, C3AR1, RNH1, NAV1, PRR16, LRRC55, ALOX12, VAV3, EPHB6, HES2, CD59, FJX1, LINC01168, ERO1A, NACAD, LOC105370969, LGALS3, DENND2D, MOCS1, NEURL1B, HS6ST1,*

		NUDT4, VCAM1, EN2, SRPX, PRODH, NIPAL1, ESR1, MMP17, CTNND1, WFIKKN2, CASS4, LPAR3, ADCYAP1R1, CFAP65, DHH, MOCOS, LGI2, PDE1C, PEAR1, PLEKHA2, CCDC102B, DIPK1C, TGFB1, MYC, TENT5C, EDNRB, CDC14B, MYO3A, OAF, PLEKHG4B, ZDHHC1, FOXS1, LOX, BNC1, TP53AIP1, SHANK2, KCNG2, FOXC2, SPSB1, MYOSLID, C6orf223, SFXN3, SLC8A1, STAT5A, MAP3K8, MTNR1B, MYO5B, CLIC2, GSX1, FAM110B, KLF9, SLC9A9, FYB1, EPB41L1, CIT, AFAP1L1, CREB5, LRFN1, TNFSF4, EOGT, TBX18, SSUH2, PRDM16, MS4A15, LINC01278, GPR157, AFP, VWA2, TMEM37, DUSP22, LOC101929130, FRMD4A, TNFRSF21, MBOAT1, OTOP2, TDO2, MARC2, RAB32, USH1G, HOPX, OPHN1, SOCS2-AS1, TMEM51, MOBP, EDNRA, ERFE, SPPL3, TSPAN18
	Down-regulated	*TRIM55, CDH6, EGR2, BMPR1B, NEFM, METTL7B, APLN, FBXO32, MAP6, GPR55, LRRC55, CMKLR1, DUSP4, TAGLN, LRRC75A, PTX3, GAP43, DUSP2, CD52, ANGPT1, NRGN, SCD5, GNG11, MATN2, CPLX2, C5, RARB, C2orf92, LOC283731*
CONTR-SH	Up-regulated	*SERPINE1*
	Down-regulated	-
SH-DAPT	Up-regulated	*FOXA2, PDZRN3, FOXJ1, SPOCK2, INSRR, PTCH1, GABRG3, SYNDIG1, LINC00261, RELN, TFAP2B, VWA5A, SLC22A8, DEPTOR, FREM1, NKX3-2, STARD4-AS1, PLCXD3, PDGFD, REC8*
	Down-regulated	*AHNAK2, TNFRSF10A, APLN, CD70, ANK1, FOSL1, FBXO32, LINC00678, SERPINE1, EMP1, CDKN1A, IL6R, ST14, IL11, CAV1, CCR7, VAT1L, TNFSF9, AEN, HES2, DUSP5, TRIM22, TNFRSF12A, CXCL14, CD44, TAGLN, TMEM132B, ATAD3B, COL5A3, LGALS3, HERC5, ZNF385A, ANXA1, PTX3, INPP5D, SMS, MTHFD2, TFPI2, OLIG3, NECTIN4, TNFRSF10D, MAFF, CDCP1, NEFM, F2RL1, PHLDA3, TRIML2, LRRC15, THBS2, LIF, HES4*
